# The rise of 3-d single-ion magnets in molecular magnetism: towards materials from molecules?

**DOI:** 10.1039/c5sc03224e

**Published:** 2015-12-23

**Authors:** Jamie M. Frost, Katie L. M. Harriman, Muralee Murugesu

**Affiliations:** a Department of Chemistry and Biomolecular Sciences , University of Ottawa , Ontario , Canada K1N 6N5 . Email: m.murugesu@uottawa.ca ; Fax: +1 613-562-5170 ; Tel: +1 613-562-5800 ext. 2733

## Abstract

Single-molecule magnets (SMMs) that contain one spin centre (so-called single-ion magnets) theoretically represent the smallest possible unit for spin-based electronic devices. The realisation of this and related technologies, depends on first being able to design systems with sufficiently large energy barriers to magnetisation reversal, *U*_eff_, and secondly, on being able to organise these molecules into addressable arrays. In recent years, significant progress has been made towards the former goal – principally as a result of efforts which have been directed towards studying complexes based on highly anisotropic lanthanide ions, such as Tb(iii) and Dy(iii). Since 2013 however, and the remarkable report by Long and co-workers of a linear Fe(i) system exhibiting *U*_eff_ = 325 K, single-ion systems of transition metals have undergone something of a renaissance in the literature. Not only do they have important lessons to teach us about anisotropy and relaxation dynamics in the quest to enhance *U*_eff_, the ability to create strongly coupled spin systems potentially offers access to a whole of host of 1, 2 and 3-dimensional materials with interesting structural and physical properties. This perspective summarises recent progress in this rapidly expanding sub-genre of molecular magnetism from the viewpoint of the synthetic chemist, with a particular focus on the lessons that have so far been learned from single-ion magnets of the d-block, and, the future research directions which we feel are likely to emerge in the coming years.

## Abstract 2

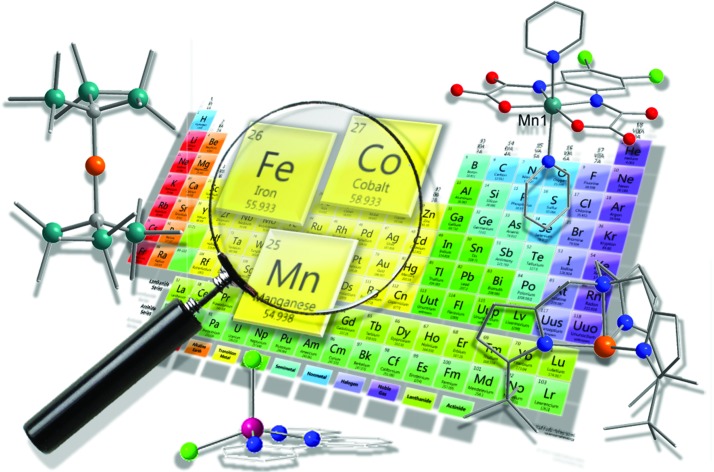
Single-molecule magnets (SMMs) that contain one spin centre (so-called single-ion magnets) theoretically represent the smallest possible unit for spin-based electronic devices. These molecules hold the promise to revolutionize computing and change the methodology by which we store, employ and process information.

## 1. Introduction: the “why”?

**Fig. 1:**
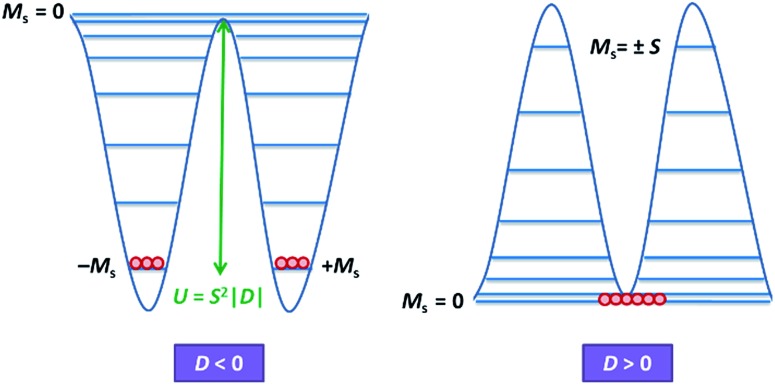
Double-well energy diagram for negative (left) and positive (right) *D*.

Magnetic materials occupy a prominent place in our daily life; from information storage technology to communication devices and medical equipment, to name but a few. Traditionally, demand for these applications has been met by rare-earth intermetallics such as SmCo_5_ and Nd_2_Fe_14_B, which are amongst the most powerful magnets known to date.^1^ However, an increasing drive towards miniaturisation of technology, combined with volatile lanthanide markets and concern surrounding the geopolitics of the rare-earth supply chain, have necessitated the exploration of new approaches to the design of smaller and cheaper alternatives. One possible method of achieving this would be to take a molecular ‘‘bottom-up’’ approach to the design of magnetic materials. Single-molecule magnets (SMMs) are molecules, which exhibit slow relaxation of their magnetisation of purely molecular origin. When a complex exhibits such behaviour, but contains only a single metal ion, they are often referred to as single-ion magnets (SIMs). Or, alternatively, mononuclear single-molecule magnets (MSMMs).^2^ We advance no argument in favour of the use of either term, it is strictly for the sake of clarity and readability that we have chosen to adopt the former. These systems continue to be at the forefront of nanomagnetic materials research and have been proposed for use in a variety of applications, including molecular spintronics,^3^ high-density information storage,^4^ and qubits for quantum information processing.^5^ Practical applications aside there is an inherent academic interest in the study of such materials, with SMMs/SIMs representing ideal model systems with which to discover and probe fascinating new physics, particularly at the interface between the classical and quantum regimes.^6^ SMMs/SIMs are superparamagnets, which display magnetic hysteresis below their blocking temperature (*T*_B_). These materials are magnetically bi-stable, exhibiting an energy barrier to spin reversal from +*M*_s_ to –*M*_s_.^7^ This concept is best illustrated using a double-well potential energy diagram, where the two wells represent the lowest energy ± *M*_s_ levels (Fig. 1). The nature of the energy barrier separating the two wells continues to be the subject of debate within the molecular magnetism community (*vide infra*), but nevertheless is often quoted as *U*_eff_ = *S*^2^|*D*| and *U*_eff_ = (*S*^2^ – ¼)|*D*| for integer and non-integer spin systems respectively.^8^ In these equations, *S* is the total spin of the complex and *D* is the axial zero-field splitting parameter, which can be positive or negative. The former describes a system in which the smallest *M*_s_ states are lower in energy than the larger *M*_s_ states; the latter, where the largest *M*_s_ states are lowest in energy. With some notable exceptions, SMMs are characterised by the presence of a negative value of *D*. When *D* is negative, the energy difference between *M*_s_ = 0 and *M*_s_ = ±*S*, denoted *U*, represents an energy barrier to thermal inversion of the magnetic moment. This means that if the thermal energy of a system (*K*_B_*T*) is less than *U*, the system will be unable to randomly reorientate its magnetic moment and will thus remain trapped in a potential energy minimum. Under such circumstances, if the system is magnetised under an applied field, upon removal of this field it can retain this magnetisation (provided *K*_B_*T* never becomes greater than *U*). This gives rise to a magnetic hysteresis effect at low temperatures of purely molecular origin, which is the defining feature of a SMM/SIM.

**Fig. 2:**
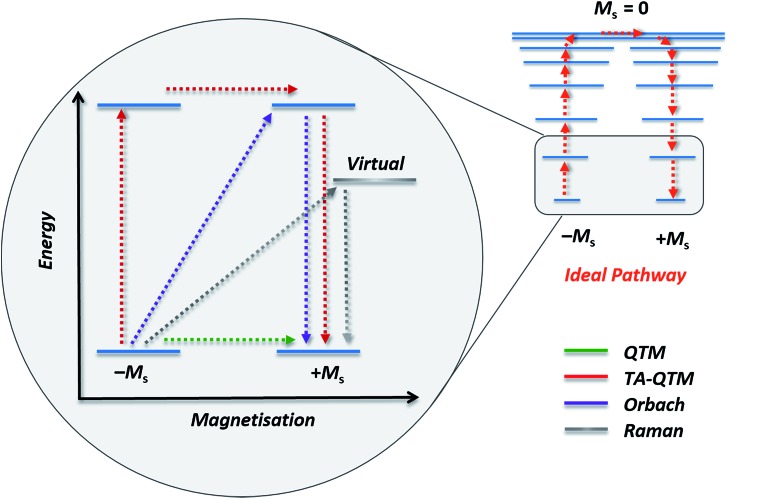
Schematic representation of possible relaxation pathways in SMMs. Blue lines represent spin states. The grey line represents a virtual state by which Raman relaxation proceeds. Colour code: green = ground state QTM, red = thermally assisted QTM (TA-QTM), purple = Orbach relaxation, grey = Raman relaxation.

The magnitude of the energy barrier to relaxation of the magnetisation in SMMs is normally determined by temperature dependent alternating current (ac) susceptibility measurements. In simple terms, the inability of the magnetisation of a given system to follow progressively larger oscillating magnetic fields is indicative (but not conclusive proof) of some energy barrier to relaxation of the magnetisation. This manifests itself as frequency dependent signals (*χ*′_m_ and *χ*′′_m_) in the in-phase and out-of-phase components respectively, of the ac susceptibility. Because the peak maximum in *χ*′′_m_ is the temperature at which the angular frequency (*ω*) of the oscillating magnetic field is equal to the rate of spin reversal (1/*τ*), the experiment is effectively a source of kinetic data and permits construction of a simple plot based on an Arrhenius rate law. For a thermally activated process over a single energy barrier a plot of ln(1/*τ*) *vs.* (1/*T*) should be linear according to the following relationship:
1

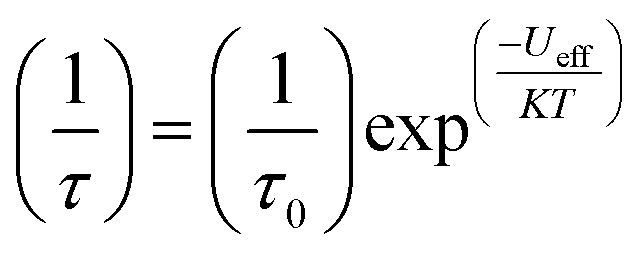

where *τ*_0_ is the relaxation rate. Of course, implicit in its use is the assumption that the system under study exhibits Arrhenius physics and that the relaxation observed arises solely from a thermal process. This is rarely the case, particularly for polymetallic systems in the weak exchange limit, and the inadequacy of eqn (1) in capturing the rich physics of SMMs is well documented in the literature.^9^ The intricacies of relaxation dynamics is a specialist topic outwith the scope of this current perspective. In simple terms though, it is helpful to think of SMM systems as being composed of two parts, the spin system and the lattice system, with interactions between spin and lattice vibrations (phonons) offering additional relaxation pathways to the system, which ‘‘shortcut’’ the thermal one. It is these additional relaxation pathways (Fig. 2) that cause experimentally observed deviations from linearity in Arrhenius plots.^10^ Specifically, there are three types of spin-lattice relaxation mechanism: (i) direct processes involve relaxation from –*M*_s_ to +*M*_s_ with emission of a single lattice phonon (ii) an Orbach process involves absorption of a phonon followed by phonon emission and relaxation from an excited state whereas (iii) a Raman process, is analogous to the Orbach mechanism with the exception that the relaxation occurs from a virtual state. SMMs can also exhibit quantum tunnelling of the magnetisation (QTM) if there is transverse anisotropy in the system – which is introduced by distortions from purely axial symmetry (for which QTM is formally forbidden).6*a* Here, the magnetisation tunnels through the anisotropy barrier between superposed ground *M*_s_ states, with tunnelling between excited *M*_s_ states possible *via* thermal/phonon assisted mechanisms. The acute sensitivity of tunnelling processes to changes in molecular symmetry, is one of the principal motivations behind the desire of synthetic chemists to control coordination number, geometry and therefore the molecular symmetry of SIMs/SMMs. In addition to these tools, and the use of magnetic dilution (*vide infra*), one common sense approach for minimising QTM through the ground state is to utilise a Kramers ion (odd electron count), for which breaking of the *M*_s_ degeneracy and thus QTM is formally forbidden in strictly zero-field.6*a*

## 2. A shift in focus: from single-molecule to single-ion magnets

Since the birth of SMM chemistry there has been a clear evolution in the focus and direction of research activity. Early studies focussed principally on high nuclearity d-block and then f-block systems with large spin ground states, whereas recent developments focus on single-ion systems of the f and d-block elements. The primary motivation behind this research evolution has been the quest to understand and control the magnetic anisotropy of single-ions, leading to higher values of both *U*_eff_ and *T*_B_. An SMM system with a *T*_B_ above room temperature is widely regarded as the holy grail of molecular magnetism. In theory, this would allow molecule-based devices to surpass conventional magnetic storage media in terms of thermal stability with respect to magnetisation decay. The first example of what we now call an SMM was a Mn(iii) cluster; [Mn_12_O_12_(OAc)_16_(H_2_O)_4_]·2MeCO_2_H·4H_2_O, often referred to simply as [Mn_12_OAc]. Magnetically characterised by Caneschi *et al.* in the early 90's (and synthesised by Lis some 11 years previously),^11^ this molecule set the benchmark for SMMs with an *S* = 10 ground state, *D* = –0.5 cm^–1^, *U*_eff_ = 60 K and *T*_B_ ≈ 3 K.^4^ Given the immense interest in [Mn_12_OAc], many synthetic chemists pursued the synthesis of new SMM compounds with a particular focus on polymetallic clusters of Mn(iii) – the Jahn–Teller (JT) distortion in the Mn(iii) ion, d^4^, largely responsible for the anisotropy of such molecules. Between the early 90's and the mid 2000's the number of reported compounds exhibiting SMM behaviour surged, and had come to encompass polymetallic systems of V, Mn, Fe, Co and Ni as well as a limited number of heterometallic 3d–4f systems.^12^ Despite rapid growth in the number of reported SMMs, progress towards increasing *T*_B_ and *U*_eff_ remained slow; the record breaking system as of 2006, [Mn^III^_6_O_2_(Et-sao)_6_(O_2_CPh(Me)_2_)_2_(EtOH)_6_], synthesised by Brechin and co-workers possessing a *T*_B_ ≈ 4.5 K and *U*_eff_ = 86.4 K.^13^ By this point however, the assumption that the development of more efficient SMMs required clusters with large total spin values, an assumption which had directed synthetic efforts towards high nuclearity clusters, was already under challenge. Several theoreticians pointed out that the oft-quoted relationship *U*_eff_ = *S*^2^|*D*|, obscures a fundamental connection between *S* and *D*, namely that *D* itself is inversely proportional to *S*^2^.^8^ In other words, incorporating large numbers of paramagnetic centres into a molecule may be counterproductive in terms of generating large cluster anisotropies (*D*_cluster_). In part, this effect can be thought of as structural; as the nuclearity of a cluster increases it becomes increasingly difficult, if not impossible, to exert control over the mutual alignment of anisotropy axes – the mutual cancellation of local anisotropies thus leading to small values of *D*_cluster_. This of course is not the whole story. For example, even in the approximately isostructural [Mn^III^_3_O(R-sao)_3_(X)(sol)_3–4_] (where R = H, Me, ^*t*^Bu; X = O_2_CR (sao = salicylaldoxime, R = H, Me, Ph *etc.*); sol = py and/or H_2_O) family of SMMs, *D*_cluster_ values of the ferromagnetically coupled *S* = 6 analogues are measurably smaller than the antiferromagnetically coupled *S* = 2 ones.^14^ This is just one select example of large magnetic anisotropy not being favoured by a high spin ground state. These factors combined with the first report by Ishikawa and co-workers in 2003 of mononuclear lanthanide systems, [TBA][Pc_2_Ln], (Pc = pthalocyanine; Ln = Tb, Dy; TBA = tetrabutylammonium) exhibiting slow relaxation of their magnetisation,^15^ helped to shift focus away from polymetallic clusters to single-ion systems.

### 2.1 Why d block SIMs?

On the face of it, single-ion complexes of the first-row transition metals may appear poorly suited to the task of building high *U*_eff_ and/or high *T*_B_ systems, at least in comparison to their lanthanide counterparts. In particular they possess; (i) smaller magnetic moments, (ii) lower spin–orbit coupling constants, and perhaps most crucially, (iii) strong coupling of the d-orbitals to the ligand field can quench first-order orbital contributions to the magnetic moment. Although arguably the first d-block SIM appeared in the literature as far back as 2003 (*vide infra*), it was not until 2010 and the report of an Fe system exhibiting slow magnetic relaxation by Long, Chang and co-workers,^16^ that mainstream interest in single-ion systems of the d-block really began. Since then, there has been a growing number of first-row d-block SIM systems reported in the literature – now extending to Mn(iii), Co(ii), Ni(i)/(II) and very recently Cr(ii). SIMs represent the simplest model systems with which to probe our understanding of the physics of spin, anisotropy and magnetic relaxation in metal complexes. The study of SIMs, and the properties that dictate their behaviour, should therefore be considered a fundamental undertaking in the quest to fabricate functional nanoscale magnetic materials from the bottom-up.

**Fig. 3:**
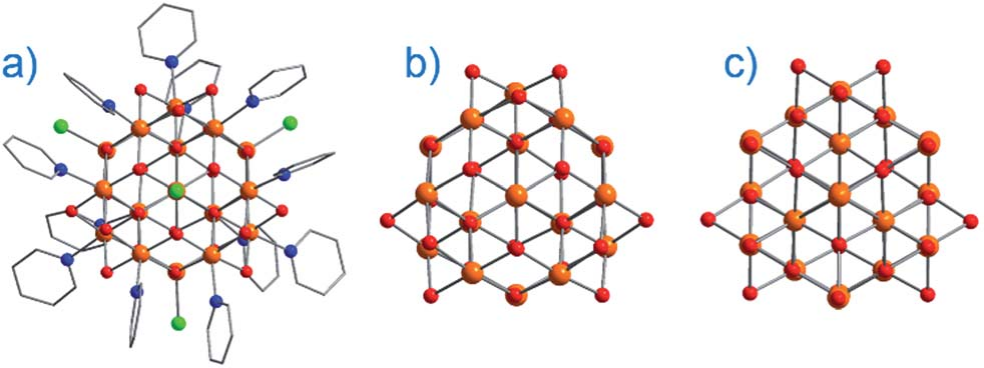
(a) Molecular structure of Hpy[Fe_17_O_16_(OH)_12_(py)_12_Cl_4_]Cl_4_ (b) metallic core of Hpy[Fe_17_O_16_(OH)_12_(py)_12_Cl_4_]Cl_4_ and (c) a portion of the structure of magnetite demonstrating its similarities with Hpy[Fe_17_O_16_(OH)_12_(py)_12_Cl_4_]Cl_4_. Colour code: orange (Fe), red (O), blue (N), green (Cl), grey (C). Hydrogen atoms omitted for clarity. Adapted from ref. 19.

The major advantage of using d-block metal ions is the ability to create strongly coupled spin systems. This is in stark contrast to the situation encountered with lanthanide ions where the core-like nature of the 4f orbitals largely prohibits this (with some notable exceptions).^17^ As we gain greater understanding of the physics of 3d single-ions in a ligand field, we can begin to develop strategies that will allow us to couple the anisotropy of individual ions together to create polymetallic systems (SMMs) in a more rational manner. Knowledge gleaned from such work can also potentially be used to revisit and improve upon the properties of existing systems. For example, synthetic chemists have long sought to create magnetically interesting molecules by targeting complexes that are fragments of known minerals.^18^ A particularly nice example of this is the Hpy[Fe_17_O_16_(OH)_12_(py)_12_Cl_4_]Cl_4_ cluster by Brechin, Collison and co-workers,^19^ whose Fe and O positions mimic a portion of the magnetite lattice (*i.e.* tetrahedral Fe(iii) sites linked to octahedral Fe(iii) ones). Capping Cl ions and py molecules occupy the peripheral metal sites, thus preventing cluster nucleation (Fig. 3). This system is not an SMM. However, if model SIM systems can be synthesised which allow a better understanding of how to extract the maximum available anisotropy from Fe ions in tetrahedral and octahedral ligand fields, then it may be possible to structurally modify these larger systems in an effort to exert control over the geometric positions of the Fe ions with respect to one another, and hence, the anisotropies of both the single ions and the resulting cluster, in an effort to engender SMM properties. Such work is invariably of relevance to those working on larger size-scale systems such as magnetic nanoparticles and bulk-magnetic materials (*e.g.* magnetite itself).

**Fig. 4:**
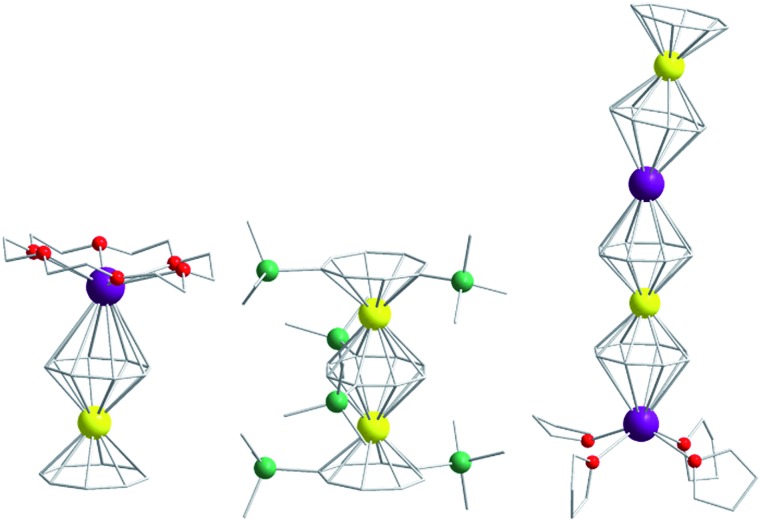
Molecular structure of some multi-decker 4f COT complexes. Colour code: yellow (Ln), purple (K), green (Si), red (O), grey (C). Hydrogen atoms omitted for clarity.

**Fig. 5:**
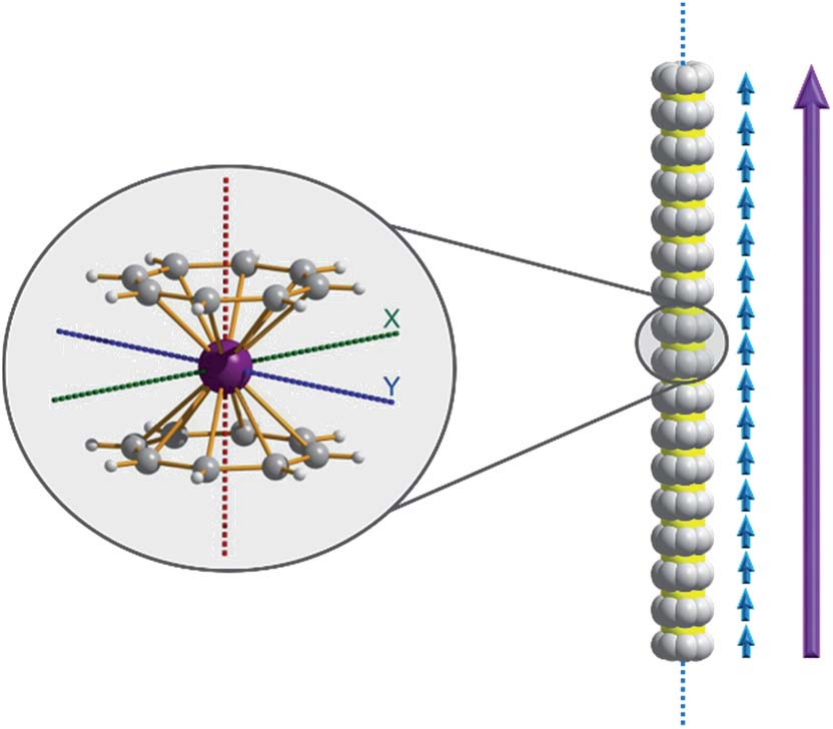
A hypothesised chain-like arrangement of M(COT)_2_ monomers with uniaxial anisotropy, illustrating the concept of modular design of single-chain magnets. The axial anisotropy of each monomer is depicted as blue vectors and the vector addition of the monomeric axial anisotropies yields the net axial anisotropy (purple).

Of course, d-block SIMs should not just be viewed as academic curiosities and model systems for polymetallic clusters, they open up new branches of chemistry in their own right. For example, the utilisation of SIM building blocks in the modular design of materials is a research area, which is already active in the 4f arena. The systematic synthesis of multi-decker cyclooctatetraenyl (COT^2–^) complexes of Gd(iii), Er(iii) and Dy(iii) is a conceptual illustration of this design principle in action (Fig. 4).^20^ Of course, the 4f ions in these structures are not strongly coupled (*J* = –0.007 to –0.48 cm^–1^ in the –2*J* formalism) to one another, and the slow relaxation behaviour of these systems can primarily be ascribed to the single-ion properties of the lanthanide ions. However, one can easily imagine that the application of similar design principles to SIM systems of the d-block ions, could result in the isolation of magnetic wires (*i.e.* single-chain magnets) (Fig. 5) with large spin and uniaxial anisotropies resulting from the coupling of single-ion properties.

Another approach would be to take SIM building blocks and assemble them into 2- and 3-dimensional networks such as metal–organic frameworks (MOFs). Not only is the ability to tune the distance of magnetic interactions between SIM units using linker ligands of varying length interesting from the magneto-chemists point of view – it constitutes one important strategy for structurally ordering SIMs to create addressable arrays for device fabrication. The synthesis of MOFs in which SIM/SMM units are used as nodes also affords a new perspective with which to approach the design of porous magnetic materials.^21^ Incidentally, SMMs have recently been incorporated into the pores of MOFs themselves, as a means of both magnetically isolating them from their surroundings, and studying the effects of host–guest interactions and confinement effects on relaxation dynamics.^22^

As one can see, the future growth and development of SIM chemistry depends first, upon a systematic exploration of synthetic factors that will allow the creation of new SIMs that can serve as suitable building blocks. Secondly, a solid understanding of the fundamental physics of these molecules, and how the various interactions, which give rise to slow relaxation can be tuned through synthetic means, is required. One subject of paramount importance in this regard is magnetic anisotropy.

## 3. Magnetic anisotropy: the key parameter?

Magnetic anisotropy is the preferential alignment of the magnetic moment along a specific direction. This normally occurs along the most energetically favourable direction of spontaneous magnetisation in a system, the so-called easy axis (the *z*-direction by definition). Or alternatively, the *xy* plane, which is denoted the easy plane. Consequently, there also exists a hard plane and a hard axis, which is the plane and axis perpendicular to the easy plane and easy axis respectively. The magnetic behaviour of SIMs is governed by the anisotropic zero-field splitting parameters, *D* and *E*, according to the following simplified Hamiltonian:

2 *Ĥ* = [*Dŝ*_*z*_^2^ – *S*(*S* + 1)/3 + *E*(*ŝ*_*x*_^2^ – *ŝ*_*y*_^2^)]

where *D* and *E* are the axial and rhombic zero-field splitting parameters, respectively, and *ŝ* is a spin operator, which describes the spin projection along a given axis. The role of *D* and *E* can be thought of as lifting the degeneracy of the 2*S* + 1 spin microstates associated with a given *S*, in the absence of an applied magnetic field. This effect is referred to as zero-field splitting (ZFS). Broadly speaking, there are two phenomena that can result in the development of ZFS and thus magnetic anisotropy: (i) first order spin–orbit coupling (in-state spin–orbit coupling) and (ii) second order spin–orbit coupling (out-of-state spin–orbit coupling).^23^ The former describes the direct mixing of spin and orbital angular momentum components in the ground electronic state of a system, whereas the latter describes the mixing of excited states, which possess first-order orbital angular momentum with the ground state, that possesses none. The magnitude of any splitting between states that results from spin–orbit coupling (SOC) is given by the spin–orbit coupling parameter, *λ*, as defined below:
3

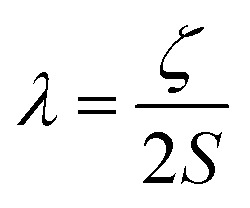

Where *ζ* is the single electron spin–orbit coupling constant and *S* is the total spin of the ion. The best way to illustrate is by example.

**Fig. 6:**
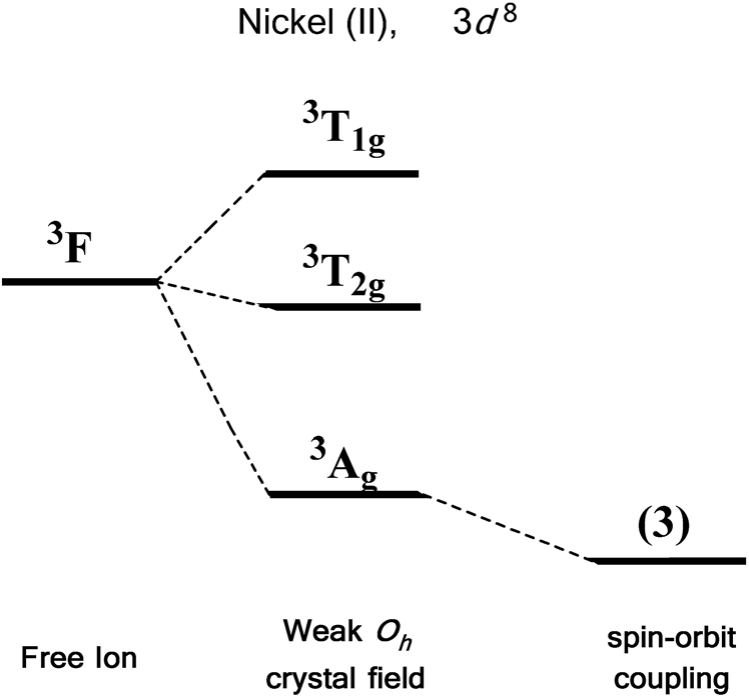
Energy level diagram illustrating the effect of a weak octahedral crystal field and spin orbit coupling on the Ni(ii) ion as described in the text. Levels expanded for clarity. The multiplicity of states arising from spin–orbit coupling are given in brackets.

We first consider the slightly simpler case of second-order SOC, using the example of Ni(ii) in an octahedral ligand field (Fig. 6). Ni(ii) is a d^8^ metal ion with a ^3^F Russell-Saunders free-ion ground term, which splits in a weak *O*_h_ field to give a ground state ^3^A_2g_ ligand field term. Although we expect no first-order SOC from an A term, the non-degenerate excited states, namely ^3^T_1g_ and ^3^T_2g_, can mix into the ^3^A_2g_ ground state. It is important to clarify that in a strictly octahedral field the result of mixing is to simply reduce the energy of the ground state term, there is no lifting of degeneracy. Of course, molecules are almost never in a perfectly symmetrical ligand field environments and it is this distortion, away from a perfect octahedral field, which lifts the degeneracy of the spin triplet ground state, thus giving rise to anisotropy. This in part helps to highlight the crucial role that symmetry plays in determining the properties of SIMs.

**Fig. 7:**
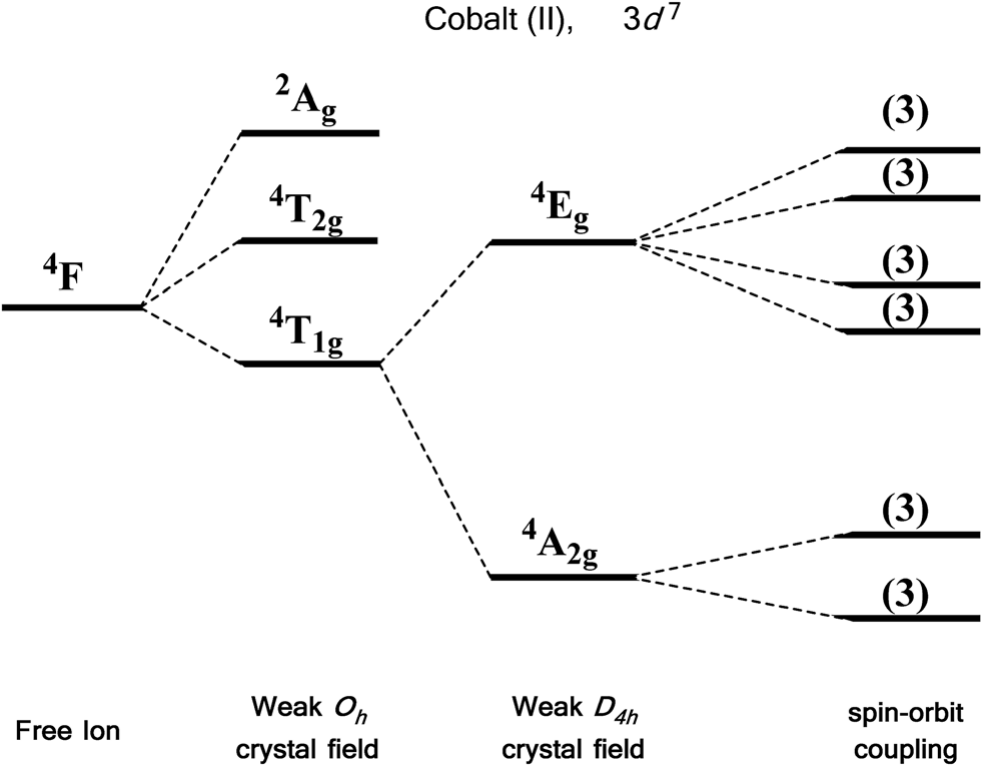
Energy level diagram illustrating the effect of a weak octahedral crystal field and spin orbit coupling on the Co(ii) ion as described in the text. Levels expanded for clarity. The multiplicity of states arising from spin–orbit coupling are given in brackets.

For an illustrative example of first-order SOC we turn to the classic case of Co(ii), a d^7^ metal with a ^4^F Russell-Saunders free-ion term (Fig. 7). In a weak octahedral field, this splits into a ^4^T_1g_ ground term with ^4^T_2g_ and ^4^A_2g_ first and second excited states, respectively. The first-order orbital angular momentum present in the ground state T term leads to a strong SOC, which splits the ground term further into a doublet, a quartet and a sextet. Again, strictly octahedral environments are rarely observed in real systems. A commonly encountered coordination geometry for Co(ii) is the axially distorted octahedron (*D*_4h_). This symmetry reduction for example splits the ^4^T_1g_ ground term into ^4^A_2g_ and ^4^E_g_ terms, which are then split by SOC into a total of six Kramers doublets, thus leading to a system, in theory, which is strongly anisotropic.

## 4. A cautionary note

Without careful attention paid to its use, the term single-molecule magnet/single-ion magnet can quickly become devoid of meaning. For example, there is an increasing trend in the literature of studying the dynamic susceptibility of systems under applied dc fields, in order to suppress QTM, which is otherwise strong for systems in low symmetry crystal environments. If such systems do not exhibit hysteresis in zero-field (in other words if there is an absence of coercivity), then strictly speaking there is a debate to be had about whether or not they can correctly be classified as magnets. This issue is particularly topical given several recent reports of SMM systems with staggeringly enormous values of *U*_eff_ (some as high as 900 K), but no corresponding coercivity in magnetisation *vs.* field studies.^24^ Of course, the application of weak dc fields can be a vital tool in elucidating the mechanisms involved in relaxation processes, however, it is important that researchers remain cautious in their interpretation of results. The application of large fields to suppress QTM invariably promotes intermolecular interactions (*vide infra*). This is particularly an issue for dried or solvent free samples whose structures may already be different to those solved by X-ray crystallography, and, those samples that have been exposed to excessive mechanical stress (*i.e.* grinding) in preparation for magnetometry studies.^25^ In the absence of careful and rigorous characterisation then, doubt can be cast on whether or not the magnetic properties of these systems are truly molecular in nature, and not simply a consequence of interactions in the solid state. Further problems arise when using high-frequency ac fields *i.e.* frequencies up to 10 000 Hz. Again, if such high frequencies need to be applied in order to observe any meaningful response from a system, the term SMM/SIM may not be entirely appropriate. A number of recent studies have been reported where such experimental conditions have been employed.^26^

The recent rise in popularity of using large applied dc fields and/or high-frequency ac fields, in our mind, rather points to the need for a debate about the precise meaning of the terms single-molecule and single-ion magnet, and where the limit lies in reasonably being able to apply such labels. This perspective however is not the appropriate medium for such a discussion. It is simply for the sake of clarity and to aid the uninitiated reader in their analysis, that we highlight these points and choose to make a clear distinction between systems that exhibit field-induced *vs.* non field-induced SIM behaviour. With this in mind, we begin with a discussion of Fe-based systems grouped according to coordination number. This seems the natural way to structure the perspective given that coordination number determines the geometry of a complex, which in turn has important consequences for the strength of the magnetic anisotropy of d-block metal ions.

## 5. Fe-based single-ion magnets

### 5.1 Five- and six-coordinate Fe-based single-ion magnets

Whilst in theory mononuclear transition metal complexes should possess high axial symmetry in order to maximise magnetic anisotropy, there are reports of lower symmetry complexes that exhibit SIM properties, even in the absence of a dc field bias. One such example is the five-coordinate Fe(iii) complex, [(PNP)FeCl_2_] (PNP = *N*[2-P(CHMe_2_)_2_-4-methylphenyl]_2_) (**1**), which also exhibits spin crossover (SCO) from *S* = 5/2 to 3/2 below 80 K.^27^ The *S* = 3/2 ground state was verified using Mössbauer and EPR spectroscopy, in addition to dc and ac magnetisation measurements. These spectroscopic measurements support the reported structural change in the molecule, which accompanies the spin transition. This change strongly influences the admixture of electronic states, in-turn affecting the spin ground state of the complex and, by extension, the observation of slow relaxation. In particular, the authors attribute the SIM behaviour, *U*_eff_ = 46 K (See Table 1), to a quantum mechanically mixed ground state comprising the *S* = 3/2 and *S* = 5/2 excited states. They suggest it is one of the principle reasons for the absence of significant QTM in zero field.

**Table 1:** Compilation of the compounds discussed in the text

Coordination number/compound	SIM, *H* = 0	SIM, *H* ≠ 0	*U*_eff_/K	*τ*_o_/s	Ref.
**Five- and six-coordinate Fe**					
[(PNP)FeCl_2_] (**1**)	Yes	—	46	2.0 × 10^–8^	27
[Fe(1-ptz)_6_](BF_4_)_2_ (**2**)	No	2000 Oe	21.6	4.2 × 10^–8^	28

**Four-coordinate Fe**					
K[(tpa^Mes^)Fe] (**3**)	No	1500 Oe	60.4	2.0 × 10^–9^	29
Na[(tpa^*t*-Bu^)Fe] (**4**)	No	1500 Oe	93.5	6.7 × 10^–11^	31
Na[(tpa^Ph^)Fe] (**5**)	No	1500 Oe	36	—	31
PhB(MesIm)_3_Fe–N=PPh_3_ (**6**)	No	1000 Oe	21.6	8.7 × 10^–7^	32
[*η*^5-5^CpFe(C_6_H_3_^*i*^Pr_3_-2,6)] (**7**)	No	750 Oe	40.3	6.0 × 10^–6^	32
		2500 Oe	143.45	7.8 × 10^–9^	

**Three-coordinate Fe**					
[Fe(N(TMS)_2_)_2_(PCy_3_)] (**8**)	No	600 Oe	23	1.6 × 10^–6^	34
[(cAAC)_2_FeCl] (**9**)	No	500 Oe	32.2	7.0 × 10^–8^	35

**Two-coordinate Fe**					
[(cAAC)_2_Fe][B(C_6_F_5_)_4_] (**10**)	No	3000 Oe	< 29	—	35
Fe[N(SiMe_3_)(Dipp)]_2_ (**11**)	No	500 Oe	260.4	1.0 × 10^–11^	35
[Fe(C(SiMe_3_)_3_)_2_] (**12**)	No	500 Oe	210.1	4.0 × 10^–9^	35
Fe[N(H)Ar′]_2_ (**13**)	No	1800 Oe	156.8	5.0 × 10^–9^	35
Fe[N(H)Ar*]_2_ (**14**)	No	875 Oe	149.6	4.0 × 10^–8^	35
Fe(OAr′)_2_ (**15**)	No	2500 Oe	61.9	3.0 × 10^–7^	35
Fe[N(H)Ar^#^]_2_ (**16**)	—	—	—	—	35
[K(crypt-222)][Fe(C(SiMe_3_)_3_)_2_] (**17**)	Yes	—	325	1.3 × 10^–9^	38

**Six and higher-coordinate Co**					
[Co(SCN)_2_(4-dzbpy)] (**18**)	Yes	—	89	2.3 × 10^–10^	39
[HNEt_3_][Co^II^Co^III^_3_L_6_] (**19**)	Yes	—	109.06	1 × 10^–7^	42
[Co(Pzox)_3_(BC_6_H_5_)]Cl (**20**)	Yes	—	102	—	43
		1500 Oe	145.3	—	
[Co^III^Co^II^(LH_2_)_2_(Cl)(H_2_O)](H_2_O)_4_ (**21**)	No	1000 Oe	11.37	6.1 × 10^–6^	44
[Co^III^Co^II^(LH_2_)_2_(Br)(H_2_O)](H_2_O)_4_ (**22**)	No	1000 Oe	20.86	1.0 × 10^–6^	44
[Co(12C4)_2_](I_3_)_2_ (12C4) (**23**)	No	500 Oe	24.5	1.5 × 10^–6^	46

**Five-coordinate Co**					
[(ArN=CMe)_2_(NPh)]Co(NCS)_2_ (**24**)	No	2000 Oe	15.97	3.6 × 10^–6^	47
[(ArN=CPh)_2_(NPh)]Co(NCS)_2_ (**25**)	No	2000 Oe	24	5.1 × 10^–7^	47
[Co(terpy)Cl_2_] (**26**)	No	600 Oe	28	1.1 × 10^–6^	48
		5600 Oe	4	7.4 × 10^–2^	
[Co(terpy)(NCS)_2_] (**27**)	No	600 Oe	16.97	5.9 × 10^–6^	48
		5600 Oe	3	0.11	
[Co(phen)(DMSO)Cl_2_] (**28**)	No	1000 Oe	10.4	5.69 × 10^–9^	49

**Four-coordinate Co**					
(Ph_4_P)_2_[Co(SPh)_4_] (**29**)	Yes	—	30.35	1.0 × 10^–6^	50
(Ph_4_P)_2_[Co(OPh)_4_] (**30**)	No	1400 Oe	30.35	7 × 10^–10^	51
K(Ph_4_P)_2_[Co_0.06_Zn_0.94_(OPh)] (**31**)	Yes	—	48.9	1.0 × 10^–9^	51
(Ph_4_P)_2_[Co(SePh)_4_] (**32**)	Yes	—	27.48	3 × 10^–6^	51
(Ph_4_P)_2_[Co(C_3_S_5_)_2_] (**33**)	Yes	—	48.78	4.5 × 10^–6^	52
[Co(quinolone)_2_I_2_] (**34**)	No	—	—	—	53
[Co(PPh_3_)_2_I_2_] (**35**)	No	1000 Oe	44.02	4.65 × 10^–10^	53
[Co(AsPh_3_)_2_I_2_] (**36**)	No	1000 Oe	46.9	1.5 × 10^–8^	53
[Co(hpbdti)_2_] (**37**)	No	2000 Oe	15.25	1.3 × 10^–5^	54
[Co(L^1^)_2_] (**38**)	No	400 Oe	49.06	7.5 × 10^–8^	54
		1000 Oe	89.06	1.0 × 10^–10^	
[Co(L^3^)_2_] (**39**)	No	400 Oe	42	1.4 × 10^–7^	54
		1000 Oe	63	2.6 × 10^–9^	
[Co(PPh_3_)_2_Br_2_] (**40**)	No	1000 Oe	39.99	5.9 × 10^–11^	54
[Co(PPh_3_)_2_Cl_2_] (**41**)	No	1000 Oe	37.12	1.2 × 10^–9^	54
[Co(DPEphos)Cl_2_] (**42**)	No	1000 Oe	34.96	2.1 × 10^–10^	54
[Co(Xantphos)Cl_2_] (**43**)	No	1000 Oe	29.92	6.0 × 10^–9^	54
[Co(P(S)([N(CH_3_)N=CHCH_3_N_2_H_3_]_3_)](NO_3_)_2_ (**44**)	No	2000 Oe	3.3	4 × 10^–6^	55
K(Co(N[CH_2_C(O)NC(CH_3_)_3_]_3_)) (**45**)	No	1500 Oe	12.52	8 × 10^–6^	

**Three-coordinate Co**					
[Li(15-crown-5)][Co(N(SiMe_3_)_2_)_3_] (**46**)	No	800 Oe	23.1	3.5 × 10^–7^	56
[Co(N(SiMe_3_)_2_)_2_(THF)] (**47**)	No	600 Oe	26	9.3 × 10^–8^	56
[Co(N(SiMe_3_)_2_)_2_(PCy_3_)] (**48**)	No	750 Oe	27.48	3.0 × 10^–7^	56

**SIMs of other metals**					
[Mn(5-TMAM(*R*)-salmen)(H_2_O)Co(CN)_6_] (**49**)	No	4500 Oe	16.55	2.9 × 10^–7^	57
Ph_4_P[Mn(opbaCl_2_)(py)_2_] (**50**)	No	1000 Oe	18.1	1.2 × 10^–7^	58
[Mn{(OPPh_2_)_2_N}_3_] (**51**)	No	2250 Oe	11.94	0.5 × 10^–7^	59
Na_5_[Mn(l-tartrate)_2_] (**52**)	No	5000 Oe	14.4	6.4 × 10^–6^	60
[Ni(6-Mes)_2_]Br (**53**)	No	600 Oe	16.98	4.6 × 10^–6^	61
[Ni(pydc)(pydm)] (**54**)	No	2000 Oe	21.2	3.83 × 10^–7^	62
[Ni(MDABCO)_2_Cl_3_][ClO_4_] (**55**)	No	500 Oe	25.2	4.1 × 10^–8^	63
		1000 Oe	27.1	2.8 × 10^–8^	
		2000 Oe	27.8	3.1 × 10^–8^	
[Cr(N(TMS)_2_)_2_(py)_2_] (**56**)	No	1500 Oe	9	1.4 × 10^–5^	64
[Cr(N(TMS)_2_)_2_(THF)_2_] (**57**)	No	2500 Oe	11.8	2.7 × 10^–6^	64

**SIMs with easy-plane anisotropy**					
[(3G)CoCl](CF_3_SO_3_) (**58**)	No	1500 Oe	34.5	1.9 × 10^–9^	65
[CoL^3^Cl_2_] (**59**)	No	2000 Oe	18.7	3.12 × 10^–7^	66
*cis*-[Co(dmphen)_2_(NCS)_2_] (**60**)	No	1000 Oe	23.3	4 × 10^–7^	67
[Co(dmphen)(Br)_2_] (**61**)	No	1000 Oe	32.9	3.7 × 10^–10^	68
[Co(abpt)_2_(tcm)_2_] (**62**)	No	3000 Oe	85.7	1.4 × 10^–9^	69
[(L^2^)_4_Co_3_(H_2_O)_2_](NO_3_)_4_ (**63**)	No	1000 Oe	8	1.0 × 10^–5^	70
[(L^1^)_4_Co_3_(H_2_O)_2_(NO_3_)_4_] (**64**)	—	—	—	—	70
[Co(L)(OAc)Y(NO_3_)_2_] (**65**)	No	1000 Oe	22.58	8.9 × 10^–7^	71
[Co(acac)_2_(H_2_O)_2_] (**66**)	No	Various	23	—	72
[Co(DAPBH)(NO_3_)(H_2_O)] (**67**)	No	Various	50–55	—	73
[Ni{^*i*^Pr_2_P(Se)NP-(Se)*^i^*Pr_2_}_2_] (**68**)	—	—	—	—	74

**Fig. 8:**
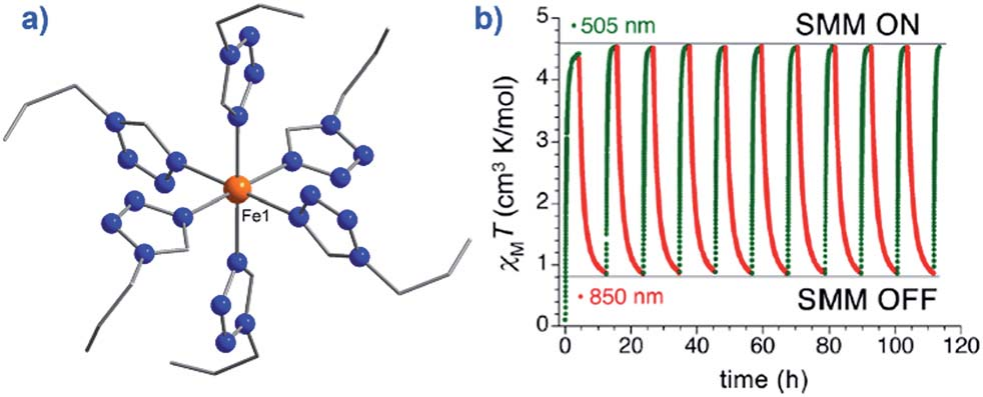
(a) Molecular structure of [Fe(1-ptz)_6_][(BF_4_)]_2_ (**2**). Colour code: orange (Fe), blue (N), and grey (C). Hydrogen atoms omitted for clarity. (b) Excitation and de-excitation cycling between the HS and LS configuration of **2**, represented as a variation in the *χ*_M_*T* product at 10 K, under a 5000 Oe field. Reprinted with permission from ref. 28. Copyright (2013) American Chemical Society.

An interesting application recently proposed for a specific polymorph of the compound [Fe(1-ptz)_6_][(BF_4_)_2_] (where ptz is propyltetrazole) (**2**), *U*_eff_ = 32 K in a 2000 Oe dc field, is ternary information storage (Fig. 8).^28^ This would involve combining the SCO properties of the Fe(ii) compound with the slow magnetic relaxation observed for the high-spin (HS) species. Light irradiation would induce a spin transition from *S* = 0 to *S* = 2, an applied dc field would result in a polarised *S* = 2 state (*i.e.* either *M*_s_ = +2 or *M*_s_ = –2). It is important to note that field-induced SIM properties are desirable here, since the applied field is the stimulus for the experimentally observed slow magnetic relaxation.

### 5.2 Four-coordinate Fe-based single-ion magnets

**Fig. 9:**
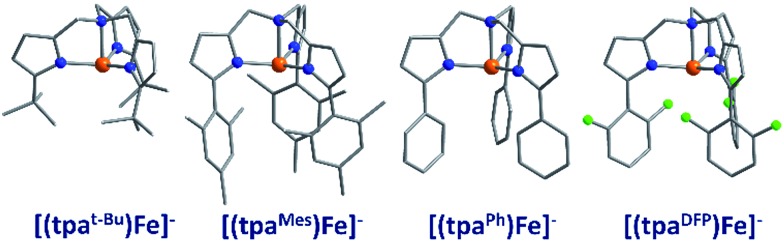
Molecular structures of four coordinate trigonal pyramidal Fe(ii) complexes of the form [(tpa^R^)Fe]^–^. Colour code: orange (Fe), green (Cl), blue (N), grey (C). Hydrogen atoms omitted for clarity.

The first reported mononuclear SIM based on a transition metal ion was the four-coordinate trigonal pyramidal complex, [(tpa^Mes^)Fe] where tpa = tris(pyrrolylmethyl)amine (**3**) (Fig. 9). *U*_eff_ = 60.4 K, albeit in an applied dc field of 1500 Oe.^29^ The complex has a large uniaxial magnetic anisotropy (*D* = –9.6 cm^–1^), which, in theory, should result in a very high thermal energy barrier. However, QTM in zero field was found to be the dominant relaxation pathway, attributed to the presence of significant transverse anisotropy (*E*). In a separate computational study, the influence of structural distortions away from ideal trigonal pyramidal geometry on the *D* value of this complex was probed. Naturally, these results can be considered a model for similar complexes.^30^ It was found that *D* decreases with large structural distortions whilst *E* increases, leading to lower energy barriers for spin reversal. A detailed *ab initio* study focusing on the magnetic anisotropy in a series of four-coordinate trigonal pyramidal Fe(ii) complexes, [(tpa^R^)Fe], structurally analogous to the aforementioned compound has also been reported (**4**, **5**).^31^

The structural distortions observed in this series, were attributed to vibronic enhancement of low-symmetry perturbations due to the R substituent of the tpa ligand. Moreover, a correlation was found between the Lewis basicity of the tpa^R^ ligands and the calculated value of *D* for each complex. This observation has important implications for the design of ligand systems, for isolating SIMs with targeted properties.

Another example of a four-coordinate Fe(ii) SIM is the phosphoraniminato-based complex [PhB(MesIm)_3_Fe–N=PPh_3_] (**6**).^32^ The system exhibits SCO behaviour and a photoactive LS (*S* = 0) to HS (*S* = 2) transition below 20 K, with continuous light irradiation below 5 K giving rise to the onset of frequency dependent ac signals. The relaxation time of the system is at its maximum in a 1000 Oe dc field yielding *U*_eff_ = 21.6 K. The ability to use ligand design strategies to simultaneously tailor both the photomagnetic properties and magnetisation dynamics of systems is an avenue ripe for future exploration.

The organometallic complex, [*η*^5-5^CpFe(C_6_H_3_*^i^*Pr_3_-2,6)] (**7**), which is highly air sensitive, is also a field-induced SIM. *U*_eff_ = 40.3 K in an applied dc field of 750 Oe increasing to *U*_eff_ = 143.45 K for a 2500 Oe dc field. Fitting of the dc magnetisation data yields a best fit with *D* = –51.36 cm^–1^ and *E* = –0.32 cm^–1^. The authors suggest the presence of significant QTM is responsible for the absence of SIM behaviour in zero-field.^33^

### 5.3 Three-coordinate Fe-based single-ion magnets

**Fig. 10:**
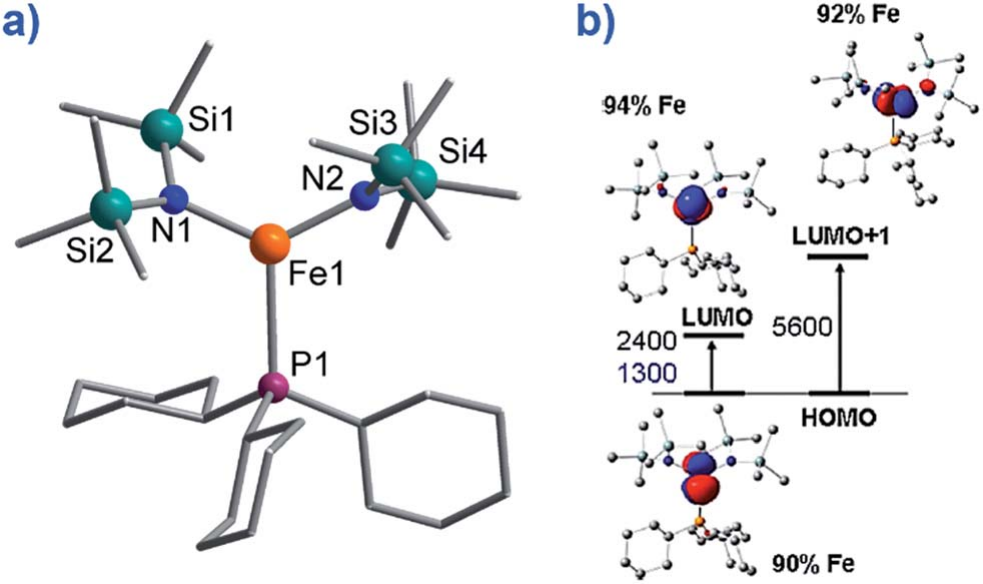
(a) Molecular structure of [Fe(N(TMS)_2_)_2_(PCy_3_)], (TMS = SiMe_3_, Cy = cyclohexyl) (**8**). Colour code: orange (Fe), plum (P), teal (Si), blue (N), grey (C). Hydrogen atoms omitted for clarity. (b) TD-DFT calculated excited states and β-spin molecular orbitals (MOs). The energies of the excited states and the metal contribution to the MOs are also shown. Reprinted with permission from ref. 34. Copyright (2011) American Chemical Society.

To the best of our knowledge only two, three-coordinate Fe complexes have been reported thus far exhibiting SIM behaviour. [Fe^II^(N(TMS)_2_)_2_(PCy_3_)] (TMS = SiMe_3_, Cy = cyclohexyl) (**8**) (Fig. 10) is one.^34^ The energy barrier for spin reversal was calculated as *U*_eff_ = 42 K under an applied field of 600 Oe. Time-dependent density functional theory (TD-DFT) calculations revealed two low lying excited states, with the corresponding molecular orbitals being principally metal-based (primarily d_*x*^2^–*y*^2^_ and d_*yz*_ character). However, these orbitals are non-degenerate and as a consequence first-order SOC is not possible. As such, one can imagine that the ZFS and in turn the slow relaxation dynamics observed in this complex, are a consequence of purely second-order SOC.

A three-coordinate cyclic alkyl(amino) carbene stabilised Fe(i) complex, [(cAAC)_2_FeCl] (**9**), has been prepared and magnetically characterised by Dalal *et al.*^35^ Mössbauer spectroscopy confirms the presence of Fe(i) with the authors ascribing the rather broad spectrum to the radical character of the *S* = ½ carbene ligand. Simulation of variable-temperature variable-field magnetisation data affords *D* = –20.4 cm^–1^ for *g* = 2.57, with complementary theoretical calculations in good agreement (*D* = –19.8 cm^–1^ for *g* = 2.54). The system exhibits rather broad frequency dependent peaks in the out-of-phase component of its ac susceptibility, below 4.1 K in an applied field of 500 Oe. Fitting to an Arrhenius rate law yields *U*_eff_ = 32.2 K. It should be noted that the presence of a radical ligand means that some may not strictly consider this system to be a SIM. In the same publication the authors also presented a two coordinate linear Fe(i) system, [(cAAC)_2_Fe][B(C_6_F_5_)_4_] (**10**).^35^ The authors indicate a *U*_eff_ = <29 K in a 3000 Oe dc field (10 times lower than the energy barrier reported for the linear complex [Fe(C(SiMe_3_)_3_)_2_] (**12**, *vide infra*). Unfortunately, the absence of data points at higher frequencies means that the authors refrain from quoting a *τ*_o_ value.

### 5.4 Two-coordinate Fe-based single-ion magnets

**Fig. 11:**
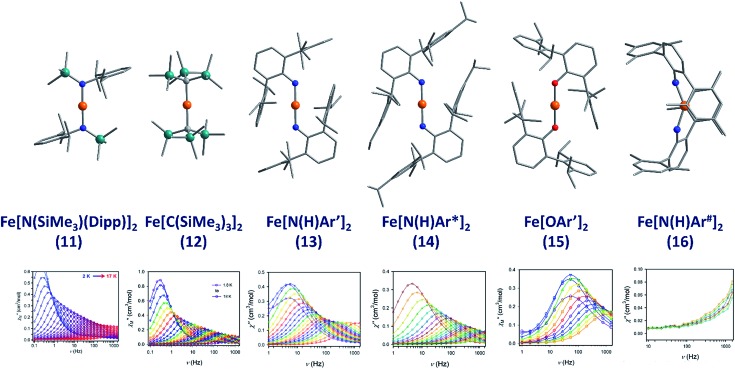
Molecular structures of linear two coordinate Fe(ii) complexes (from left to right): Fe[N(SiMe_3_)(Dipp)]_2_ (**11**), Fe[C(SiMe_3_)_3_]_2_ (**12**), Fe[N(H)Ar′]_2_ (**13**), Fe[N(H)Ar*]_2_ (**14**), Fe[OAr′]_2_ (**15**), and Fe[N(H)Ar^#^]_2_ (**16**). Colour code: orange (Fe), teal (Si), blue (N), red (O), grey (C). Hydrogen atoms omitted for clarity. Below is the frequency dependence of the out-of-phase magnetic susceptibility (*χ*′′) as a function of temperature for each complex. Data were collected under applied dc fields of 500 Oe (**11**), 500 Oe (**12**), 875 Oe (**13**), 875 Oe (**14**), 2500 Oe (**15**), and 1000 Oe (**16**), respectively. Reprinted with permission from ref. 36*b*. Copyright (2013) Royal Society of Chemistry.

The lowest coordinate Fe-based complex thus far reported that exhibits SIM properties has been two-coordinate with a linear geometry about the metal centre. The main goal of lowering the coordination number of a 3d metal ion is to mitigate ligand-field effects, which otherwise quench orbital contributions to the magnetic moment, thus reducing anisotropy. In addition, the magnitude of *D* is also inversely proportional to the energy gap between ground and excited states. Therefore, ensuring that the energies of the d-orbitals fall within a narrow range of one another facilitates better mixing of ground and excited states potentially leading to larger *D* values. With this consideration in mind, a series of homoleptic Fe(ii) complexes have been prepared by Long and co-workers, exhibiting rigorous linear geometry with local *D*_h_ symmetry at the metal ion.^36^ By carefully varying the ligand field strength around the Fe(ii) centres, they show how it is possible to increase the observed magnetic anisotropy. For the ligand field here, the d-orbitals are split such that their energies follow the order (d_*xy*_, d_*x*^2^–*y*^2^_) < (d_*xz*_, d_*yz*_) < d_*z*^2^_. The δ symmetry of the first group of orbitals with respect to the axial ligands indicates that that they are non-bonding in nature. A d^6^ ion in the HS state with this specific coordination geometry will exhibit strong anisotropy due to the δ_g_ set of orbitals, which are triply occupied, resulting in significant first-order contributions to the orbital angular momentum. Hence, by modulating the ligand field, the authors were able to create a series of complexes with a range of *D* values and, by extension, spin reversal barriers. The complexes, Fe[N(SiMe_3_)(Dipp)]_2_ (**11**), Fe[C(SiMe_3_)_3_]_2_ (**12**), Fe[N(H)Ar′]_2_ (**13**), Fe[N(H)Ar*]_2_ (**14**) and Fe(OAr′)_2_ (**15**) (Fig. 11) all behave as SIMs under an applied dc field with *U*_eff_ = 260, 210, 156.8, 149.6 and 61.9 K, respectively. One other complex, Fe[N(H)Ar^#^]_2_ (**16**), with bent geometry about the Fe(ii) centre exhibits only the tails of frequency dependent peaks in ac susceptibility measurements. This is because the bent structure creates a large splitting between the lowest lying d-orbitals (d_*xy*_, d_*x*^2^–*y*^2^_), and thus strong quenching of orbital angular momentum results. This lends credence to the proposal that strict linear geometries are necessary for the development of electronic configurations which yield highly anisotropic *g*-tensors. The lack of SIM properties in the absence of an applied dc field is attributed to the presence of significant QTM mediated by transverse anisotropy (*E*), nuclear hyperfine coupling and/or dipolar interactions.

In order to gain more insight into the electronic and magnetic properties of these two-coordinate Fe(ii) systems, theoretical calculations were carried out by Atanasov *et al.*^37^ The lowered symmetry and splitting of the ground state (^5^Δ) was attributed to the interplay between; (i) strong mixing of the 3d_*z*^2^_–4s orbitals for all complexes, (ii) σ–π type orbital mixing for the Fe–O bonds, and (iii) π-bonding anisotropy due to the strongly π-donating amide ligands in the amide containing complexes. Based on these calculations, the authors developed several guidelines for the synthesis of SIMs with improved relaxation times. These include; (a) replacing C, N, or O donor atoms with their heavier analogues Si, P and S in order to minimise vibronic coupling and increase SOC; (b) choosing metal–ligand bonds with high local pseudo-symmetry such as *C*_3v_ or *C*_2v_; (c) minimising secondary metal–ligand interactions by utilising bulky ligands with aliphatic moieties as opposed to aromatic substituents and (d) minimising dipolar spin–spin interactions between metal centres using either distance, magnetic dilution or deposition on surfaces. The authors also point out that strategies to suppress QTM should be adopted wherever possible. QTM is of course particularly efficient for these systems due to the small non-Kramers *S* = 2 ground state, an attributing factor to the absence of slow relaxation in zero-field.

A chemically reduced, two-coordinate linear complex, [Fe(C(SiMe_3_)_3_)_2_]^–^ (**17**), has been synthesised by Long *et al.* as a proof of principle, where the Fe centre is in the +1 oxidation state with an *S* = 3/2 spin ground state.^38^ Computational analysis yielded an energy splitting of the 3d orbitals that was unexpected: d_*z*^2^_ < (d_*xy*_, d_*x*^2^–*y*^2^_) < (d_*xz*_, d_*yz*_). This favours large magnetic anisotropy leading to SIM behaviour in the absence of an applied field with *U*_eff_ = 325.2 K, the largest yet reported for a transition metal SIM and, quite astonishingly, starting to approach values seen in lanthanide-based systems.

Fe has established itself as an ideal candidate for building SIM systems. The highlight of this growing body of Fe literature has to be the linear Fe(i) compound (**17**) with *U*_eff_ = 325.2 K, which stands out not only because of the enormous energy barrier to relaxation but also because of the synthetic ingenuity, which was required to isolate such a molecule. Without a doubt, there remains many more interesting low coordinate Fe(i) compounds waiting to be made. The ability to switch on SIM behaviour in Fe-based SCO compounds also raises exciting possibilities in terms of the potential applications of these molecules. In addition, given the hundreds, if not thousands, of Fe-based SCO materials characterised over the years (even before the emergence of SMM/SIM chemistry), there may exist entire libraries of dormant photo-switchable SIM compounds waiting to be re-discovered in the literature.

## 6. Co(ii)-based single-ion magnets

**Fig. 12:**
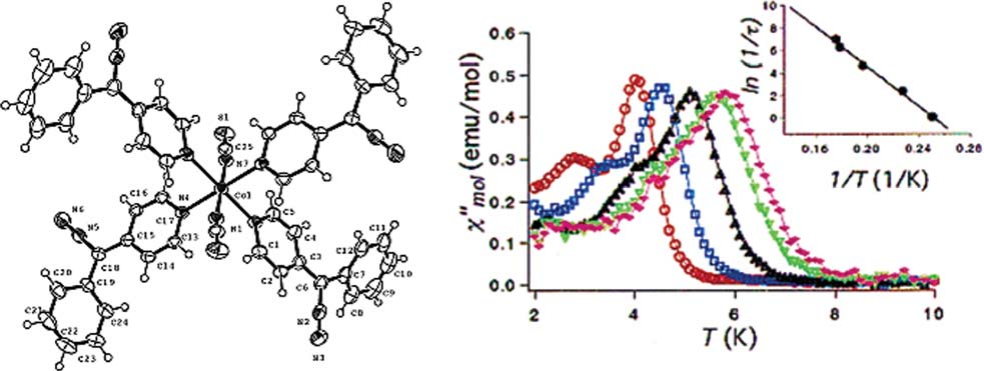
Molecular structure of [Co(SCN)_2_(4-dzbpy)_4_] (**18**) (left) and out-of-phase magnetic susceptibility as a function of temperature (right), with an inset showing the Arrhenius fit of the data. Reprinted with permission from ref. 39. Copyright (2003) American Chemical Society.

Cobalt is a good candidate for the synthesis of SIM systems due to the strong first order SOC displayed by the metal in the 2+ oxidation state. In fact, arguably, the first ever d-block SIM was a Co(ii) complex. [Co(SCN)_2_(4-dzbpy)_4_] (dzbpy is diazobenzylpyridine) was published by Koga and co-workers in 2003 (**18**) (Fig. 12).^39^ The molecule consists of a single octahedral metal centre, coordinated to four 4-dzbpy and two NCS ligands in a *trans* configuration. The goal of this work at the time was to study the magnetic properties of heterospin systems, where 3d metal centres are coordinated to carbenes with 2p spins. By coupling two spin-containing species the authors reasoned that large *D* and *S* values may be obtained, potentially leading to high barrier SIMs. The carbene was generated *in situ*, with the magnetic properties measured before and after light irradiation, to provide a reliable comparison. Frozen solution measurements indicate ferromagnetic interactions between the two spin systems after the triplet carbene is generated, as well as slow relaxation behaviour characteristic of an SIM. *U*_eff_ was reported to be 89 K. Hysteresis measurements yielded open hysteresis loops at 3.5 K confirming the SIM nature of the Co(ii)–carbene complex. It is important to note that this complex was not isolated in the carbene form; rather the precursor was isolated and single-crystal X-ray diffraction studies were performed on that. Therefore, some ambiguity exists about the exact nature of the structure which magnetic measurements were obtained for. While this was the first report of slow relaxation behaviour in a system with a single 3d ion, the metal is not the sole source of spin and hence some consider it not to be a ‘‘pure’’ SIM. Since then, other reports have surfaced combining radical ligands with 3d ions in an effort to target large barrier SIM/SMMs.^40^ Indeed, this is currently something of a hot topic in molecular magnetism, with a recent review being dedicated solely to the subject.^41^ In addition to these radical based systems, there have been several reports of mononuclear Co(ii) complexes bearing neutral ligands which exhibit SIM properties.

### 6.1 Six-coordinate and higher Co(ii) single-ion magnets

**Fig. 13:**
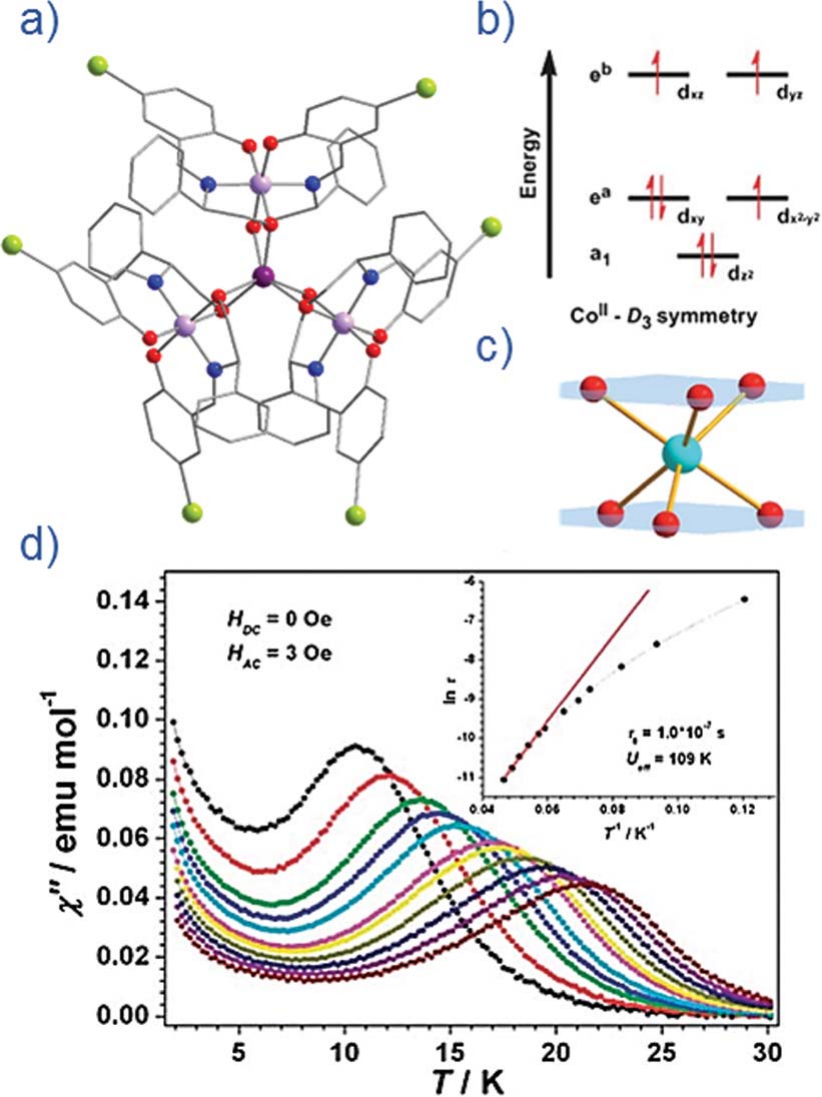
(a) Molecular structure of [HNEt_3_][Co^II^Co^III^_3_L_6_] (**19**). Colour code: dark purple (Co(ii)), light purple (Co(iii)), red (O), blue (N), grey (C) and light green (Br). Hydrogen atoms omitted for clarity. (b) Simplified d-orbital splitting diagram (c) simplified coordination environment of central Co(ii) ion and (d) Out-of-phase magnetic susceptibility for **19** under zero applied dc field. Arrhenius plot of natural log of the relaxation time *vs.* inverse temperature as inset. Reprinted with permission from ref. 42. Copyright (2013) Royal Society of Chemistry.

Thus far, the majority of mononuclear Co(ii) SIMs with axial magnetic anisotropy have been complexes with coordination numbers ≤5. Indeed, higher coordination number Co(ii) complexes typically possess dominant positive/easy plane anisotropy (see Section 8). One six-coordinate complex, reported by Gao and co-workers is [HNEt_3_][Co^II^Co^III^_3_L_6_] (**19**) where L_6_ is the Schiff-base *R*-4-bromo-2-((2-hydroxy-1-phenylethylimino)methyl)phenol (Fig. 13).^42^ The complex consists of a paramagnetic Co(ii) centre surrounded by three diamagnetic Co(iii) ions. The central Co(ii) is coordinated by six O-atoms originating from the L ligands. This produces a slightly distorted trigonal prismatic geometry (*D*_3_ symmetry). The magnetic behaviour of this compound arises solely from the Co(ii) centre and hence can effectively be considered a SIM. The ZFS parameter was calculated to be *D* = –115 cm^–1^, indicating a highly anisotropic system. Slow magnetic relaxation is observed in zero field with *U*_eff_ = 109 K, one of the highest values reported for a mononuclear Co(ii) system. The high relaxation barrier can be attributed to a very small transverse anisotropy, which reduces the influence of QTM on the thermally assisted relaxation process. Additionally, the three peripheral Co(iii) ions serve to weaken intermolecular exchange and dipolar interactions between Co(ii) centres, effectively producing a dilution-like effect.

**Fig. 14:**
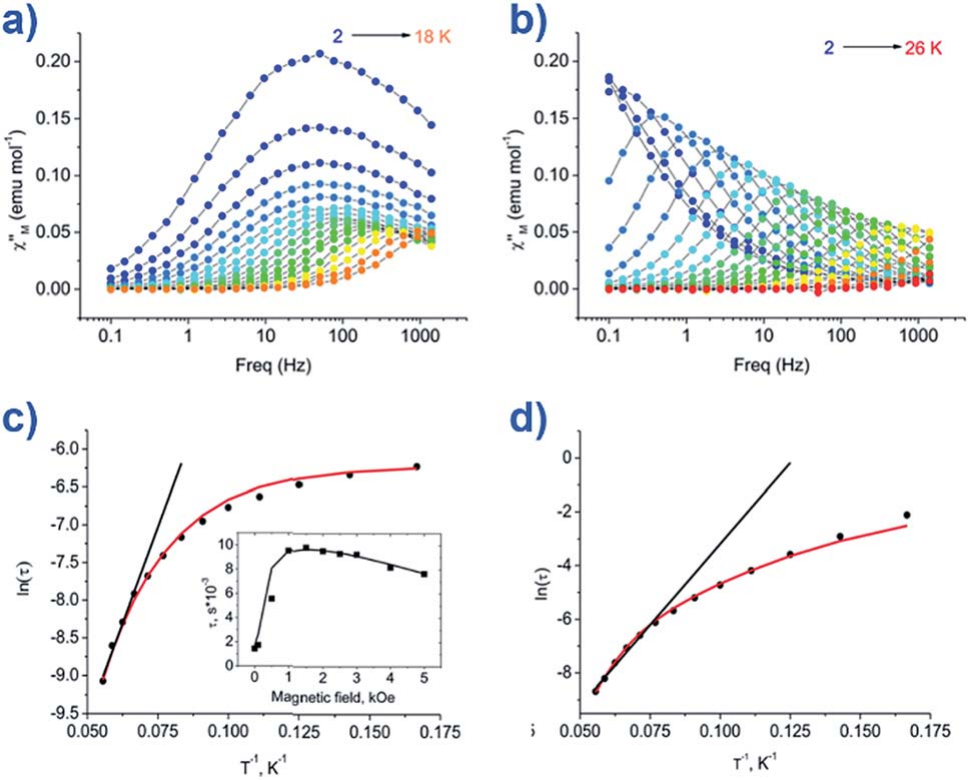
Out-of-phase magnetic susceptibility for [Co(Pzox)_3_(BC_6_H_5_)]Cl (**20**) measured under; (a) zero applied dc field and (b) an applied dc field of 1500 Oe. (c) Arrhenius plot of the natural log of the relaxation time *vs.* inverse temperature under a zero applied dc field and (d) an applied dc field of 1500 Oe. Reprinted with permission from ref. 43. Copyright (2015) American Chemical Society.

The group of Novikov very recently reported another six-coordinate Co(ii) SIM with trigonal prismatic geometry.^43^ The complex, [Co(Pzox)_3_(BC_6_H_5_)]Cl (**20**) (Fig. 14), was built utilising tris-pyrazoloximate (Pzox) ligands, chosen to provide a sufficiently weak ligand field to ensure that the Co(ii) ions are high-spin at low temperature. Dynamic susceptibility studies revealed the system to be an SIM in zero-field with *U*_eff_ = 102 K, however if a static dc field is applied (1500 Oe) an increase in the thermal energy barrier is observed (*U*_eff_ = 145.3 K). The reason for the difference between the *U*_eff_ in zero-field and in an applied field, is of course due to the different relaxation processes which are operative under these respective conditions. The authors demonstrated that quantum tunnelling dominates in the zero-field/low temperature regime, Raman relaxation is prevalent both in the presence and absence of an applied field over all temperatures, and, Orbach relaxation is evident both in the presence and absence of a field, but is only important at high temperatures. The energy barrier for the Orbach-only processes was calculated as *U* = 218.7 K, which is substantially higher than the *U*_eff_ value observed under a dc field. To the best of our knowledge, this awards the system the accolade of having the highest reported relaxation barrier of any Co(ii) SIM. The discrepancy between *U* and *U*_eff_ here highlights an important point – even in the absence of QTM, a large energy gap to the first excited state (220 cm^–1^ here) does not necessarily guarantee a large magnetisation reversal barrier. Multi-phonon Raman processes can take over when sufficiently high-energy phonons are not available. The authors suggest that this issue should be an important consideration for anyone attempting to maximise *U*_eff_ values in Co(ii)-based SIM systems. We wholeheartedly agree.

**Fig. 15:**
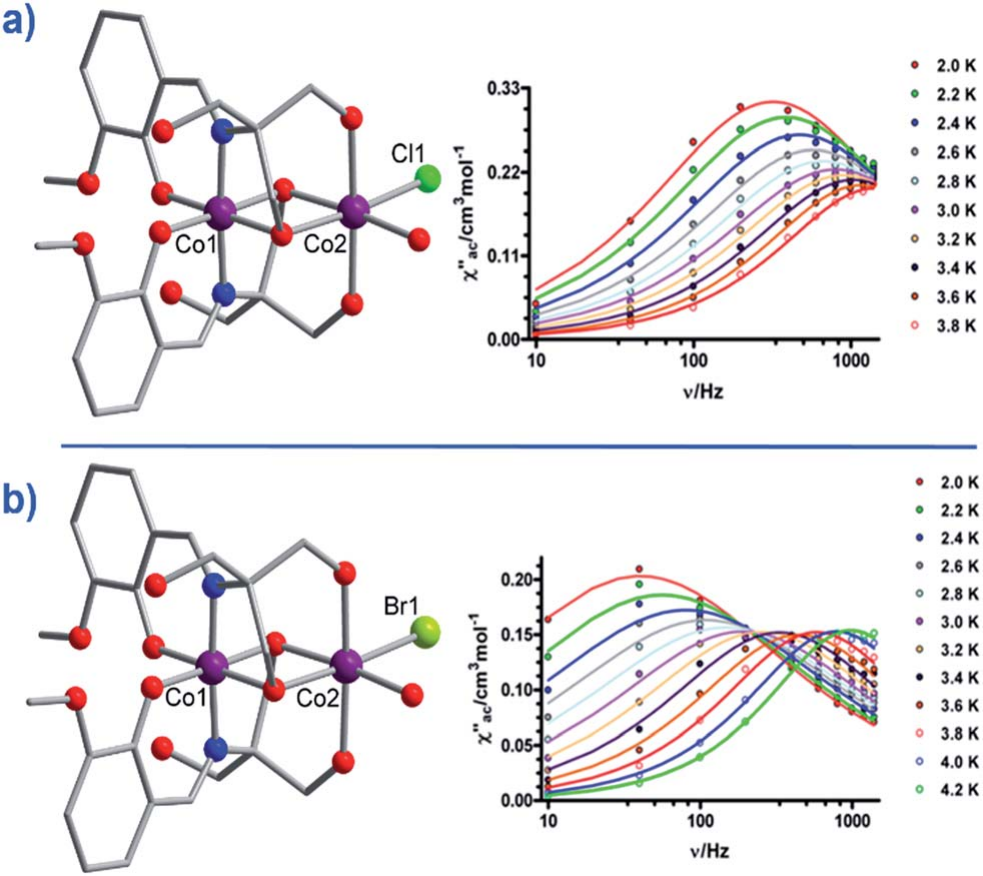
Molecular structures of [Co^III^Co^II^(LH_2_)_2_(X)(H_2_O)](H_2_O)_4_ and the frequency dependence of the out-of-phase magnetic susceptibility collected in a 1000 Oe dc field. (a) X = Cl (**21**) and (b) X = Br (**22**). Colour code: purple (Co), bright green (Cl), light green (Br), blue (N), red (O), grey (C). Hydrogen atoms omitted for clarity. Reprinted with permission from ref. 44. Copyright (2014) American Chemical Society.

Another two, six-coordinate Co(ii) SIM systems, are the dinuclear mixed-valence clusters [Co^III^Co^II^(LH_2_)_2_(X)(H_2_O)](H_2_O)_4_ (**21**, X = Cl, **22**, X = Br) prepared by Colacio and co-workers (Fig. 15) using the Schiff-base 2-[{(2-hydroxy-3-methoxyphenyl)methylene}amino]-2-(hydroxymethyl)-1,3 propane-diol.^44^ Both Co(ii) ions are in distorted octahedral geometries, with the diamagnetic Co(iii) ions effectively rendering both complexes single-ion systems in magnetic terms. Both systems reveal broad frequency dependent peaks in ac susceptibility studies with fitting of the data to an Arrhenius model yielding, *U*_eff_ = 12.52 and 20.86 K for **21** and **22** respectively, (both systems measured in a 1000 Oe dc field). The authors suggest the absence of slow relaxation in zero-field is a result of QTM, but correctly point out that this is likely mediated by hyperfine and/or dipolar interactions, since of course transverse anisotropy cannot mix the wavefunctions of ±*M*_s_ levels for non-integer spin-systems with *D* < 0 in strictly zero-field.^45^

Intriguingly, an eight-coordinate Co(ii) system has been reported to exhibit SIM properties. The complex, [Co^II^(12C4)_2_](I_3_)_2_(12C4)(12C4 = 12-crown-4)] (**23**), is the first eight-coordinate 3d mononuclear complex to show slow relaxation of the magnetisation (albeit under an applied dc field of 500 Oe), with a rather modest *U*_eff_ = 24.5 K.^46^

### 6.2 Five-coordinate Co(ii) single-ion magnets

**Fig. 16:**
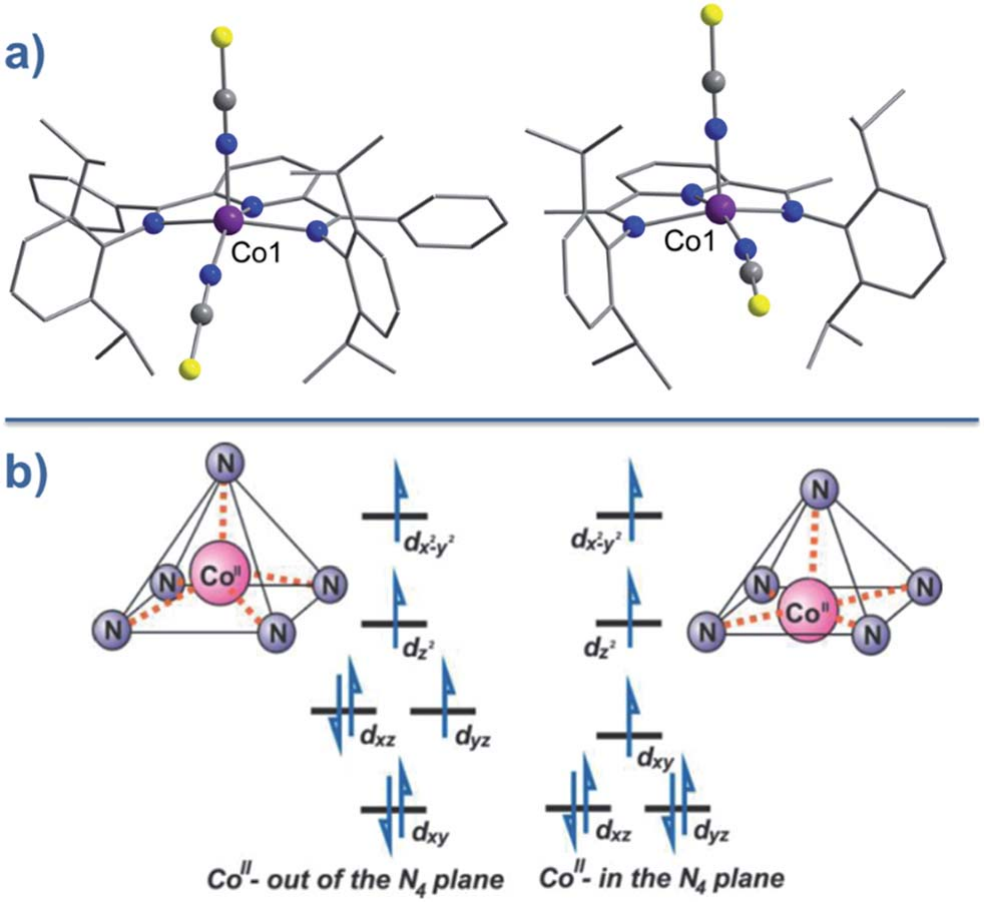
(a) Molecular structures of [Co(LPh)(NCS)_2_] (**25**, left) and [Co(LMe)(NCS)_2_] (**24**, right). Colour code: purple (Co), blue (N), yellow (S), grey (C). (b) d-orbital splitting diagrams for **24** and **25**, highlighting the effect of metal ion displacement from the basal plane. Reprinted with permission from ref. 47. Copyright (2011) American Chemical Society.

**Fig. 17:**
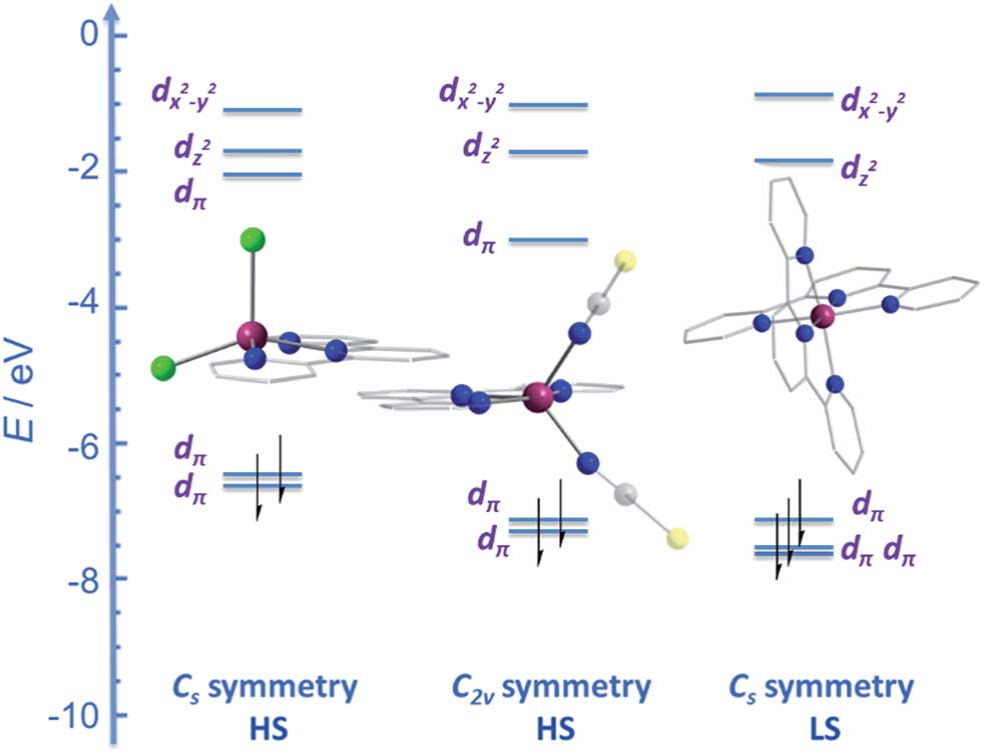
Energy level diagram depicting selected β-spin frontier molecular orbitals of [Co(terpy)Cl_2_] (*C*_s_ symmetry) (**26**), [Co(terpy)(NCS)_2_] (*C*_2v_ symmetry) (**27**) and [Co(terpy)_2_]^2+^ (*C*_s_ symmetry). The increase in the number of β-spins for [Co(terpy)_2_]^2+^ comes at the cost of an α-spin, resulting in an overall decrease in the molecular spin state. Reprinted with permission from ref. 48. Copyright (2013) Wiley-VCH.

Two of the first mononuclear Co(ii)-based SIMs; the penta-coordinate complexes [Co({ArN=CMe}_2_(NPh))(NCS)_2_] (**24**) and [Co({ArN=CPh}_2_(NPh))(NCS)_2_] (**25**) (Fig. 16), were actually reported by ourselves and our collaborator Darrin Richeson.^47^ Ligand design was crucial here in order to favour square pyramidal over trigonal bipyramidal geometry. Hence we settled on using bis(imino)pyridine pincers. The ligand was carefully designed to create tension within the basal plane, thus pushing the Co(ii) ion out-of-plane and promoting SOC. We achieved this by modifying the pincer ligands at the imine position using methyl or phenyl groups. The remaining coordination sites in these complexes are occupied by NCS ligands, chosen because they can easily accommodate distortions in metal-ion geometry. Slow magnetic relaxation was observed for both complexes, under an applied field of 2000 Oe, with *U*_eff_ = 16 and 24 K for **24** and **25** respectively. Using simple planar terpyridine (terpy) ligands we were able to prepare two more five-coordinate complexes in collaboration with Robert Crabtree; [Co(terpy)Cl_2_] (**26**) and [Co(terpy)(NCS)_2_] (**27**) (Fig. 17).^48^ These systems consist of a tridentate terpy ligand coordinated to Co(ii), with the remaining two coordination sites occupied by monodentate Cl or NCS ligands. The electronic structure of these molecules was studied using DFT, which led to the energy level diagram shown in Fig. 17. Since a HS state is necessary at low temperature in order to observe SIM behaviour (and the lower coordination number of the mono-terpy *vs.* bis-terpy (5 *vs.* 6) is expected to stabilise the HS state), we targeted the mono-terpy complexes first as opposed to the bis-terpy compounds (which we later found to exhibit SCO behaviour). TD-DFT calculations support the presence of low-lying excited states, which contribute greatly to the anisotropy of these complexes. It is noteworthy that geometry optimisation for both systems led to a complex with *C*_s_ symmetry, which is in accordance with the X-ray structure of the first complex [Co(terpy)Cl_2_]. However, for the NCS complex, X-ray crystallography reveals a structure with *C*_2v_ symmetry, indicating that crystal-packing effects may exert a strong influence on symmetry here.

Ac susceptibility measurements for both [Co(terpy)Cl_2_] and [Co(terpy)(NCS)_2_] revealed two sets of peaks in the field dependent *χ*′′ *vs. ν* plots, which interestingly, is similar to the situation encountered by Long and co-workers in their studies on tetrahedral Co(ii) complexes (*vide infra*). We found that each process is dominant under a different applied dc field. Under *H* = 600 Oe, a thermal relaxation pathway was observed at *ν* > 10 Hz, leading to *U*_eff_ = 28 and 17 K for [Co(terpy)Cl_2_] and [Co(terpy)(NCS)_2_] respectively. The second relaxation pathway was observed at *ν* < 1 Hz, in an applied field of 5600 Oe leading to *U*_eff_ < 4 K for both complexes. *Ab initio* calculations were performed to shed some light on these processes and the difference in magnetic properties between the Cl and NCS derivatives. For [Co(terpy)Cl_2_], the first excited Kramers doublet was shown to be approximately twice as high in energy compared to the NCS complex (200 cm^–1^*vs.* 100 cm^–1^). Moreover, the transverse component of the *g*-factors were shown to be relatively large for both, possibly explaining the lack of slow magnetic relaxation at *H* = 0, which arises due to quantum tunnelling *via* transverse dipolar fields. It is noteworthy that in addition to the lower first excited Kramers doublet, the transverse anisotropy was calculated to be larger in the NCS complex, which potentially explains the lower energy barrier observed for this compound in comparison to the Cl derivative.

Trávníček and co-workers recently reported another five-coordinate Co(ii) system, [Co(phen)(DMSO)Cl_2_] (**28**) (where phen = 1,10′-phenanthroline), which is also a field-induced SIM.^49^ Magnetisation studies revealed *D* = –17 cm^–1^ and *E*/*D* = 0.24, with DFT calculations lending support to these values (*D* = –17.7 cm^–1^ and *E*/*D* = 0.31 cm^–1^ by theory). Ac susceptibility data was fitted to a Debye model yielding *U*_eff_ = 10.4 K in a 1000 Oe dc field.

### 6.3 Four-coordinate Co(ii) single-ion magnets

Typically, most Co systems only show magnetic blocking under an applied dc field. That being said, a notable example of a system showing slow relaxation in the absence of an applied field is the tetrahedral complex [Co(SPh)_4_]^2^ (**29**), reported by Long.^50^ The high-spin Co(ii) ion is shown to possess an *S* = 3/2 spin ground state with large, negative, axial ZFS (*D* = –70 cm^–1^). Additionally, this complex was shown to possess relatively low rhombicity with *E*/*D* < 0.09. The large magneto-anisotropy can be studied qualitatively here by examining the d-orbital splitting of the Co(ii) ion. The filled d_*z*^2^_ orbital is calculated to be lowest in energy, followed by a filled d_*x*^2^–*y*^2^_ orbital. At slightly higher energy lies the singly-occupied d_*xy*_ orbital, which is in close enough proximity to the d_*x*^2^–*y*^2^_ orbital such that a low-lying excited electronic state is generated, which can SOC to the ground state. The last two singly-occupied 3d orbitals, d_*xz*_ and d_*yz*_, are calculated to be highest in energy. The large *D* value here results in an energy barrier for spin reversal of *U*_eff_ = 30.2 K. An interesting feature can be observed for this system in the *χ*′′ *vs. ν* plot as a function of field. As the strength of the field is increased, one relaxation process (at higher frequency) is seen to decrease in intensity whilst another (at lower frequency) appears to gain intensity. This reflects the change in the relaxation mechanisms from thermally activated (at higher frequencies) to quantum tunnelling (at lower frequencies) depending on the magnitude of the applied dc field. To further probe the change in relaxation mechanisms, magnetic dilution studies were performed using the isomorphous Zn(ii) analogue, which confirmed the molecular nature of the magnetic properties^50^*i.e.* a second relaxation process was not observed in these samples, thus indicating the intermolecular nature of the second process observed in the parent sample.

**Fig. 18:**
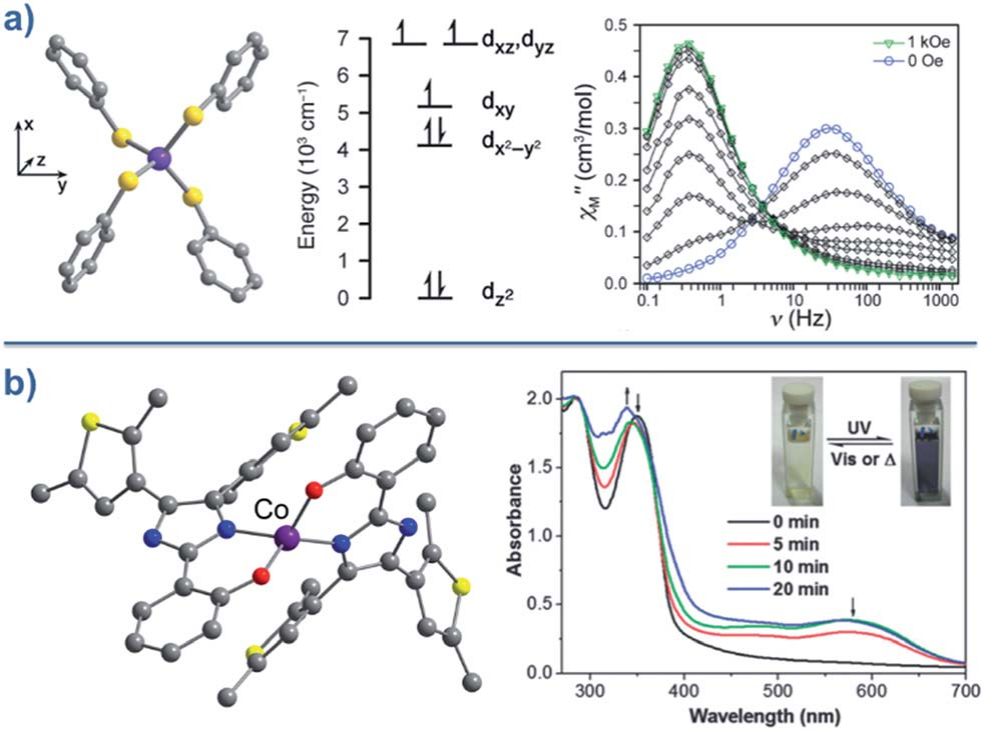
(a) Molecular structure, d-orbital splitting diagram and *χ*′′ *vs. ν* plot as a function of *H* for [Co(SePh)_4_]^2–^ (**32**). Colour code: purple (Co), yellow (S), grey (C). Counter cation and hydrogen atoms omitted for clarity. Reprinted with permission from ref. 50. Copyright (2011) American Chemical Society. (b) Molecular structure and absorption spectra changes under UV irradiation, thus demonstrating the photochromic behaviour of [Co(hpbdti)_2_] (hpbdtiH = 2-(2-hydroxpheyl)-4,5-bis(2,5-dimethyl(3-thienyl))-1*H*-imidazole) (**37**). Colour code: purple (Co), yellow (S), red (O), blue (N), grey (C). Hydrogen atoms omitted for clarity. Reprinted with permission from ref. 54*a*. Copyright (2013) Royal Society of Chemistry.

A series of complexes with the general formula [Co(EPh)_4_]^2–^ (E = O, S and Se), was later reported by the same authors (**30–32**)^51^ in order to study the relationship between *D* and the energy barrier for spin reversal. However, no clear relationship could be established since the barriers remained the same for different donor atoms. It is noteworthy that the *D* value did vary however, from *D* = –11.1 cm^–1^ in (Ph_4_P)_2_[Co(OPh)_4_] (**30**) to –83 cm^–1^ in (Ph_4_P)_2_[Co(SePh)_4_] (**32**) (Fig. 18). In these complexes the magnetic anisotropy appears to originate from a second order SOC interaction between ground and low-lying excited states. Second order SOC is also responsible for generating a very large value of *D* in the pseudo-tetrahedral complex, (Ph_4_P)_2_[Co(C_3_S_5_)_2_] (**33**) (*D* = –161 cm^–1^ with a negligible rhombic component).^52^ Again, these results highlight the importance of minimising d-orbital splitting in order to promote strong SOC. The most common sense approach for achieving this is of course to exploit weak ligand fields with soft donor atoms. This hypothesis is further supported by the recent work of Dunbar and co-workers, who prepared a series of pseudo-tetrahedral Co(ii) complexes; [Co(quinolone)_2_I_2_] (**34**) and [Co(EPh_3_)_2_I_2_] (**35–36**) (*E* = P, As).^53^ They too observed an increase in *D* using heavier main group donor atoms. Whilst the metal ions became increasingly anisotropic however, the energy barriers for spin reversal did not increase significantly either; *D* (cm^–1^)/*U*_eff_ (K) = +9.2/not quantifiable, 36.9/30.6, –74.7/32.6 for **34**, **35** and **36**, respectively. Sadly, the reason for these observations remains unclear.

Other Co(ii) complexes exhibiting distorted tetrahedral geometry have been reported where SIM behaviour is observed both in the presence and absence of an applied dc field (**37–43**).^54^

An interesting proof-of-principle recently demonstrated by Ruiz and co-workers, is the ability to computationally predict the anisotropy of d-block metal complexes based on simple considerations such as coordination geometry, symmetry around the metal ion and d-electron count. Using CASSCF calculations, two molecules already known in the literature were identified as target candidates for exhibiting SIM behaviour. Experimental measurements confirm this, with [Co(P(S)([N(CH_3_)N=CHCH_3_N_2_H_3_]_3_))](NO_3_)_2_ (**44**) and K(Co(N[CH_2_–C(O)NC(CH_3_)_3_]_3_)) (**45**) exhibiting *U*_eff_ = 33.1 and 12.52 K in 2000 and 1500 Oe dc fields respectively.55*a* This development is certainly interesting and suggests that computational chemistry has an important role to play in the future development of SIM chemistry. Not simply in rationalising the magnetic properties of newly synthesised systems (where it has already proven itself invaluable), but actually in directing the synthesis of new compounds, which do not yet exist or are magnetically uncharacterised. Such work on the part of theoreticians could rapidly accelerate progress towards the goals of increasing *U*_eff_ and *T*_B_. Indeed, although not a d-block system we note with great interest the recent report by Winpenny, Mills and co-workers of an as yet fictitious Dy(iii) linear bis(amide) complex, which *ab initio* calculations suggest should possess a staggering *U*_eff_ value of 2589 K.55*b*

### 6.4 Three-coordinate Co(ii) single-ion magnets

**Fig. 19:**
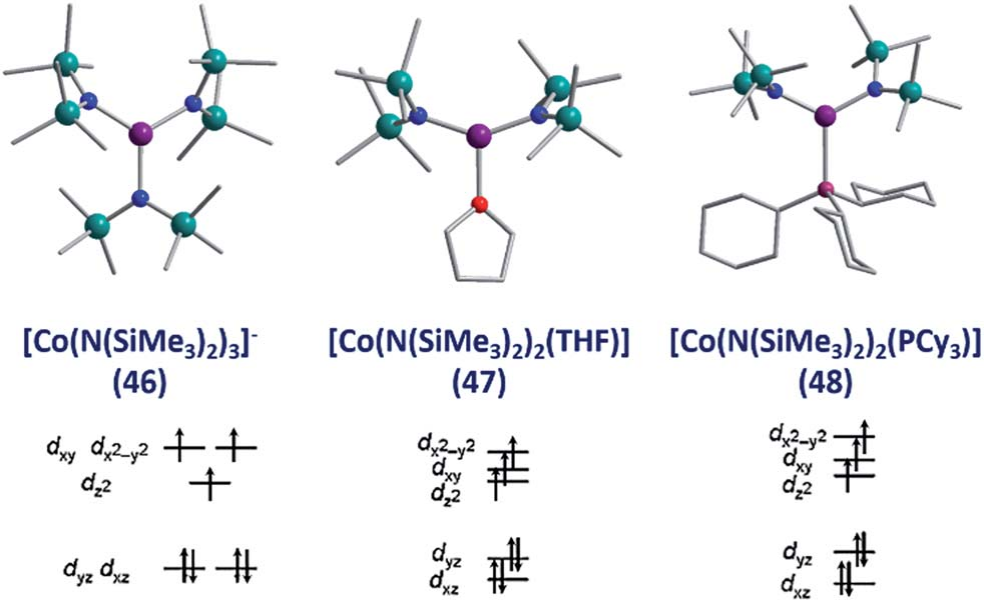
Molecular structure and d-orbital splitting diagram for the three coordinate Co(ii) complexes, [Li(15-crown-5)][Co{N(SiMe_3_)_2_}_3_] (**46**), [Co{N(SiMe_3_)_2_}_2_(THF)] (**47**) and [Co{N-(SiMe_3_)_2_}_2_(PCy_3_)] (**48**). Colour code: purple (Co), plum (P), teal (Si), blue (N), red (O), grey (C). Hydrogen atoms omitted for clarity. Adapted with permission from ref. 56. Copyright (2014) American Chemical Society.

To the best of our knowledge the only examples of three coordinate Co(ii) SIMs are the three related complexes; [Li(15-crown-5)][Co{N(SiMe_3_)_2_}_3_] (**46**), [Co{N(SiMe_3_)_2_}_2_(THF)] (**47**) and [Co(N(SiMe_3_)_2_)_2_(PCy_3_)] (**48**), published by Eichhöfer and co-workers in 2014 (Fig. 19).^56^ The three systems are strongly anisotropic with *D* = –57, –72 and –82 cm^–1^ for **46**, **47** and **48**, respectively. The complexes exhibit different *U*_eff_ values; 23.1 K (800 Oe dc field), 26 K (600 Oe dc field) and 27.48 K (750 Oe dc field) for **46**, **47**, and **48** respectively. The authors attribute these minor differences to subtle changes in the energies of the frontier d orbitals, which occur upon ligand substitution across the three molecules; from a strong σ-donor/π-donor ligand in **46** (N(SiMe_3_)_2_), to a σ-donor/weak π-donor in **47** (THF), to a weak σ-donor/weak π-acceptor in **48** (PCy_3_).

Interest in SIM systems of Co(ii) continues to flourish, due in large part to the unquenched first-order angular momentum exhibited by the ion which, in theory, can lead to large values of *D*. It is interesting to note that the vast majority of Co(ii) SIMs require an applied dc field in order to observe slow relaxation behaviour – with the exception of **19** and **29–34**. The zero-field behaviour of the former complex, [HNEt_3_][Co^II^Co^III^_3_L_6_], can likely be ascribed to both the *D* value of the central Co(ii) ion and more crucially the magnetic dilution-like effect, which is created by the surrounding Co(iii) ions. For the latter systems, as discussed, the zero-field behaviour is probably a consequence of their low coordination number and tetrahedral geometries. These observations further highlight the role the synthetic chemistry has to play in the continued development of SIM chemistry. The targeted isolation of Co(ii) SIM systems exhibiting slow relaxation in zero-field, will require a systematic exploration of the effects of ligand design, metal-ion geometry and cluster symmetry on the magnetic properties of these molecules. As Ruiz and co-workers have also demonstrated the computational chemist has an equally important part to play.

## 7. Other 3d single-ion magnets

Whilst Co(ii)- and Fe(I/II/III)-based systems have so far dominated this exciting sub-genre of molecular magnetism, there are reports of other 3d metal ion complexes exhibiting slow magnetic relaxation. These include mononuclear complexes of Mn(iii) as well as recently reported Ni(I/II) and Cr(ii) complexes.

**Fig. 20:**
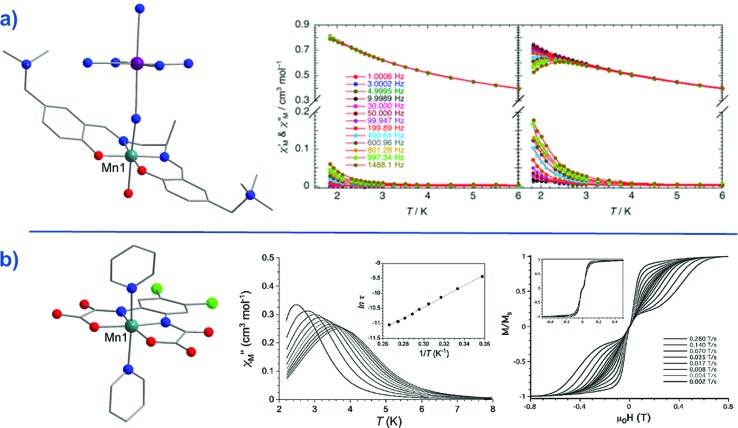
(a) Molecular structure of [Mn^III^(5-TMAM(*R*)-salmen)(H_2_O)Co^III^(CN)_6_]·7H_2_O·MeCN (**49**) Colour code: teal (Mn), purple (Co), blue (N), red (O), bright green (Cl), grey (C). Hydrogen atoms omitted for clarity. To the right of this, is the temperature dependence of the ac susceptibilities (*χ*′ and *χ*′′) under an applied dc field of 4500 Oe and an ac field of 5 Oe. Reprinted with permission from ref. 57. Copyright (2013) American Chemical Society. (b) Molecular structure of Ph_4_P[Mn(opbaCl_2_)(py)_2_] (**50**). Colour code: teal (Mn), blue (N), red (O), bright green (Cl), grey (C). Hydrogen atoms omitted for clarity. To the right of this, is the temperature dependence of the out-of-phase magnetic susceptibility (*χ*′′) under an applied dc field of 1000 Oe and 4 Oe oscillating field (inset: Arrhenius plot), and, the tweep rate dependence of the magnetisation at 0.5 K (inset: at 0.03 K). Reprinted with permission from ref. 58. Copyright (2013) Wiley-VCH.

The Mn(iii)-salen-type complex reported by Yamashita and co-workers,^57^ [Mn^III^(5-TMAM(*R*)-salmen)(H_2_O)Co^III^(CN)_6_]·7H_2_O·MeCN (**49**) (Fig. 20) (5-TMAM(*R*)-salmen = (*R*)-*N*,*N*′-(1-methylethylene)bis(5-trimethylammoniomethylsalicylidene iminate)), is not strictly mononuclear. However, the diamagnetic low-spin Co(iii) ion effectively renders the system a SIM. A Jahn–Teller distortion (axial elongation) of the Mn(iii) ion provides the necessary magnetic anisotropy to generate a spin reversal barrier (*D*_Mn_ = –3.3 cm^–1^). It is important to note that only the tails of frequency dependent peaks were observed in ac susceptibility studies, even with application of a dc field. The authors report an energy barrier of *U*_eff_ = 13.4 K.

A truly mononuclear Mn(iii) complex, Ph_4_P[Mn(opbaCl_2_)(py)_2_] (**50**) (where H_4_opbaCl_2_ = *N*,*N*′-3,4-dichloro-*o*-phenylenebis(oxamic acid), py = pyridine and Ph_4_P = tetraphenylphosphonium), was recently reported by Pardo, Armentano and Cano which exhibits SIM behaviour under an applied dc field.^58^ The authors employed the aforementioned planar tetradentate ligand in order to isolate a mononuclear complex in axially elongated octahedral geometry. Fitting of the dc magnetisation data gave *D* = –3.27 cm^–1^, *E* = –0.11 cm^–1^ and for *g* = 1.99, confirming the presence of second-order SOC. Ac magnetic susceptibility measurements revealed SIM behaviour under an applied dc field of 1000 Oe, with a calculated barrier for spin reversal, *U*_eff_ = 18 K. This is in reasonable agreement with other mononuclear 3d SMMs that are significantly affected by fast QTM. Micro-SQUID measurements revealed temperature-dependent and sweep rate-dependent butterfly-shaped hysteresis loops but no coercivity in zero field.

The groups of Sanakis and Kyritsis have reported magnetic studies on the already known complex [Mn{(OPPh_2_)_2_N}_3_] (**51**). Naturally, the central Mn(iii) ion adopts a distorted octahedral geometry, with fitting of the magnetisation data yielding *D* = –3.4 cm^–1^. Despite the *D* value however the complex only exhibits a *U*_eff_ = 11.94 K even in a 2250 Oe dc field. The authors attribute this to QTM arising from the non-zero rhombicity in the system.^59^ The mononuclear complex Na_5_[Mn(l-tartrate)_2_] (**52**) was recently found by Murrie and co-workers to exhibit *U*_eff_ = 14.4 K in a 5000 Oe dc field. HF-EPR studies reveal *D* = –3.23 cm^–1^ with a rhombic anisotropy ∼1% of *D*.^60^

In addition to Mn(iii)-based clusters exhibiting SIM properties, Whittlesey and co-workers in collaboration with ourselves, were able to demonstrate slow magnetic relaxation in a Ni(i) system.^61^ The complex, [Ni(6-Mes)_2_]Br (**53**) where 6-Mes = 1,3-bis(2,4,6-trimethylphenyl)-3,4,5,6-tetrahydropyrimidin-2-ylidene, exhibits a linear, two-coordinate geometry about the metal centre. DFT calculations performed on the complex as well as its closed shell Ni(0) analogue revealed that the Ni(i) complex has a very similar d orbital arrangement to the reduced analogue, which helps to explain the observed magnetic anisotropy. SIM behaviour with an energy barrier of *U*_eff_ = 17 K, was observed in an applied dc field of 600 Oe. Of course, since Ni(i) is a Kramers ion which should theoretically experience no QTM, SIM properties should be observed without the application of an external field. However the field-induced nature of the slow relaxation can be attributed to mixing of ground and thermally accessible excited states. Titiš and co-workers recently reported the first example of a Ni(ii) SIM, the complex [Ni(pydc)(pydm)] (**54**),^62^ (pydc = pyridine-2,6-dicarboxylate, pydm = 2,6-bis(hydroxylmethyl)pyridine), which features the ion in axially compressed pseudo-octahedral geometry. This situation results in a sizeable rhombic contribution to the magnetic anisotropy with *D*/*hc* ≈ –14 cm^–1^, and as a consequence the system only exhibits field-induced SIM behaviour under a 2000 Oe dc field, with *U*_eff_ = 21.2 K. Interestingly, ac susceptibility studies reveal evidence of two distinct relaxation processes, with the authors suggesting the first (faster) relaxation process is the single-molecule process, and the second (slower) process is the relaxation of Ni(i)–Ni(i) dimers which are held together by weak π–π interactions.

Another recent Ni(ii) system worthy of mention is the trigonal bipyramidal [Ni(MDABCO)_2_Cl_3_][ClO_4_] (**55**) complex recently characterised by Murrie and co-workers^63^ The complex only exhibits slow relaxation behaviour in the presence of a dc field bias, with energy barriers of 25.2, 27.1 and 27.8 K in dc fields of 500, 1000 and 2000 Oe respectively. The most notable feature of this complex though, is the fact that the authors suggest a *D* value of ∼–535 cm^–1^ based on HF-EPR studies, with a corresponding *E* value of <0.18 cm^–1^. The observation of such large single-ion anisotropy is attributed to the lack of axial symmetry breaking, and hence strong retention of trigonal symmetry, courtesy of the bulky MDABCO ligands. Given the impressively high *D* value reported the lack of slow relaxation behaviour in zero-field is disappointing (but perhaps not unexpected for a Ni(ii) system). Nevertheless, this result is very interesting for us, and in our opinion **55** constitutes an ideal model system, with which to develop strategies to suppress the contribution of Raman and direct relaxation processes to *U*_eff_ – the dominant pathways in this system.

Finally, It would be remiss of us not to mention the very recent report of field-induced slow relaxation in two Cr(ii) complexes.^64^ [Cr(N(TMS)_2_)_2_(py)_2_] (**56**) and [Cr(N(TMS)_2_)_2_(THF)] (**57**) (TMS = SiMe_3_) exhibit *U*_eff_ = 9 K and 11.8 K respectively under 1500 and 2500 Oe dc fields respectively. HF-EPR studies yield *D* and *E* values of –1.80 and 0.020 cm^–1^, and, –2.00 and 0.025 cm^–1^ for **56** and **57** respectively. Indeed, this represents the first observation of slow relaxation of any d-block ion in a square-planar coordination geometry, as well as the first reported example of a Cr(ii) SIM.

## 8. Single-ion magnets with easy-plane anisotropy

There are a growing number of exceptions to the generally accepted rule that SMMs require negative or uni-axial anisotropy, which it is prudent for us to mention. Several complexes have been reported in recent years to exhibit slow magnetic relaxation and SIM behaviour whilst possessing positive or easy-plane magnetoanisotropy. These mononuclear complexes are all based on Co(ii) (to the best of our knowledge) and behave as SIMs only under an applied dc field.

**Fig. 21:**
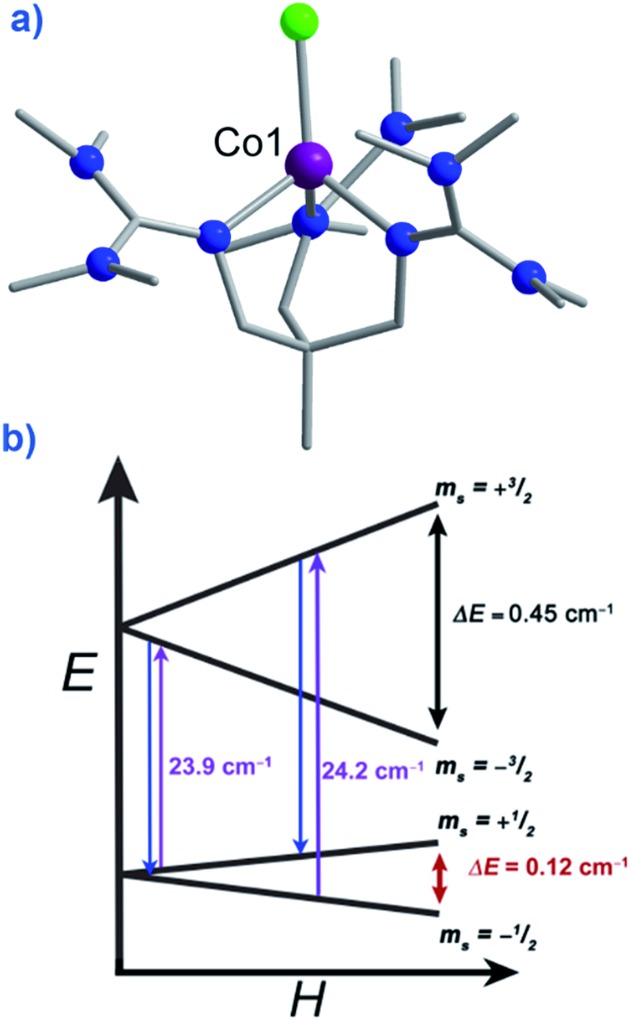
(a) Molecular structure of [(3G)CoCl] (CF_3_SO_3_) (**58**). Colour code: purple (Co), bright green (Cl), blue (N), grey (C). Hydrogen atoms omitted for clarity. (b) Zeeman splitting diagram for **58**, where the red arrows indicated the direct relaxation process, the purple arrows correspond to the excitation energies related to the Orbach processes, and the blue arrows correspond to the relaxation *via* the Orbach process. All values presented were determined for *S* = 3/2, *g*, *D*, and *E* as determined by EPR spectroscopy. Reprinted with permission from ref. 65. Copyright (2012) Royal Society of Chemistry.

Recently Long, Chang and Hill published magnetic studies on a pseudotetrahedral Co(ii) complex, [(3 G)CoCl](CF_3_SO_3_) (**58**) where 3G is 1,1,1-tris[2-*N*-(1,1,3,3-tetramethylguanidino)methyl]ethane (Fig. 21). The system has a *D* = +12.7 cm^–1^ (measured by EPR spectroscopy) with spin-lattice relaxation observed to occur between the lowest lying *M*_s_ = ±1/2 levels. This is as opposed to relaxation through the *M*_s_ = ±3/2 levels seen in analogous complexes with easy-axis (Ising-type) anisotropy.^65^ Whilst it is difficult to explain or rationalise the observation of slow magnetic relaxation in complexes where *D* > 0, the authors suggest that a phonon bottleneck is responsible for slowing down the direct relaxation process and allowing an Orbach process to occur through the higher energy *M*_s_ = ±3/2 levels. Additionally, the rhombic anisotropy term (*E*) was found to be non-zero and serves to mix the *M*_s_ = ±1/2 and *M*_s_ = ±3/2 levels of opposite sign leading to more efficient spin relaxation through the excited *M*_s_ = ±3/2 levels and an energy barrier of *U*_eff_ = 34.5 K.

A penta-coordinate Co(ii) SIM, [CoL^3^Cl_2_] (**59**) (L^3^ = 4-hept-1-ynyl-2,6-dipyrazol-l-ylpyridine),^66^ has also been reported by Boča. The aromatic tridentate ligand facilitates π–π stacking of the discrete molecules, thus forming dimers and leading to intermolecular ferromagnetic interactions between metal centres. While large magnetic anisotropy is observed (*D*/*hc* > +150 cm^–1^ and *E*/*hc* = +11.6 cm^–1^), blocking of the magnetisation is only seen under a 2000 Oe dc field, yielding *U*_eff_ ≈ 13 K. This energy barrier represents just one of the two relaxation processes observed in the field dependent ac plots.

Pardo *et al.* have reported field-induced SIM behaviour in a six-coordinate Co(ii) complex, *cis*-[Co^II^(dmphen)_2_(NCS)_2_] (**60**) (dmphen = 2,9-dimethyl-1,10-phenanthroline), with strong easy-plane magnetoanisotropy (*D* = + 98 cm^–1^ and *E* = 8.4 cm^–1^).^67^ The authors also suggest that the origin of the observed spin reversal barrier, *U*_eff_ = 24.5 K, could be governed by the magnitude of *E*. In a situation where *E* = (*D*_*xx*_ – *D*_*yy*_)/2, the transverse anisotropy would create a preferred axis for the spin along the *x* or *y* direction. When this occurs, the spin flip (from +*x* to –*x* or +*y* to –*y*) would be governed by *D* = 3*D*_*zz*_/2 if it occurs through the *z*-axis. Conversely, if it occurs through the *xy* plane, it would be controlled by the *E* parameter. According to the experimentally obtained *D* and *E* values for the distorted octahedral Co(ii) complex, the calculated energy barrier is found to correspond to a spin rotation within the *xy* plane where *U*_eff_ = 2*E*. Hence, in this case, the observed slow magnetic relaxation is dependent on the transverse anisotropy energy barrier. An extremely similar system based on the same ligand has also reported by Duan *et al.*^68^ [Co^II^(dmphen)(Br)_2_] (**61**) exhibits *U*_eff_ = 32.9 K in a 1000 Oe dc field. Dilution studies using up to 80% Zn(ii), corroborate the molecular nature of the relaxation process. Another pseudo-octahedral complex, [Co(abpt)_2_(tcm)_2_] (**62**) (abpt = 4-amino-3,5-bis(2-pyridyl)-1,2,4-triazole and tcm = tricyanomethanide), has also been reported to exhibit easy plane anisotropy, with a spin reversal barrier of *U*_eff_ = 85.7 K. This is the highest reported barrier thus far for this special sub-class of SIMs.^69^ A further example of a Co(ii) system where the authors suggest a similar mechanism is operative is to be found in [(L^2^)_4_Co_3_(H_2_O)_2_](NO_3_)_4_ (**63**) (where L^2^ is the ligand prepared from the Schiff-base condensation of pyridine-2-carboxylate and carbohydrazone).^70^ The system features a single Co(ii) ion in six-coordinate distorted octahedreal geometry, which forms the centre of a trinuclear Co^III^–Co^II^–Co^III^ array. Fitting of ac susceptibility data to an Arrhenius model yields *U*_eff_ = 8 K in a 1000 Oe dc field with the authors suggesting *D* = +31.9 cm^–1^ and *E* = –3.0 cm^–1^. It is again proposed by the authors that the non-negligible value of *E* here is crucial in allowing slow relaxation in the *xy* plane. Indeed, an analogous molecule made using a slightly modified ligand, [(L^1^)_4_Co_3_(H_2_O)_2_](NO_3_)_4_ (**64**), exhibits *D* = –18.6 cm^–1^ and *E* = –1.7 cm^–1^ and does not exhibit any frequency dependent signals in ac susceptibility studies.^70^

**Fig. 22:**
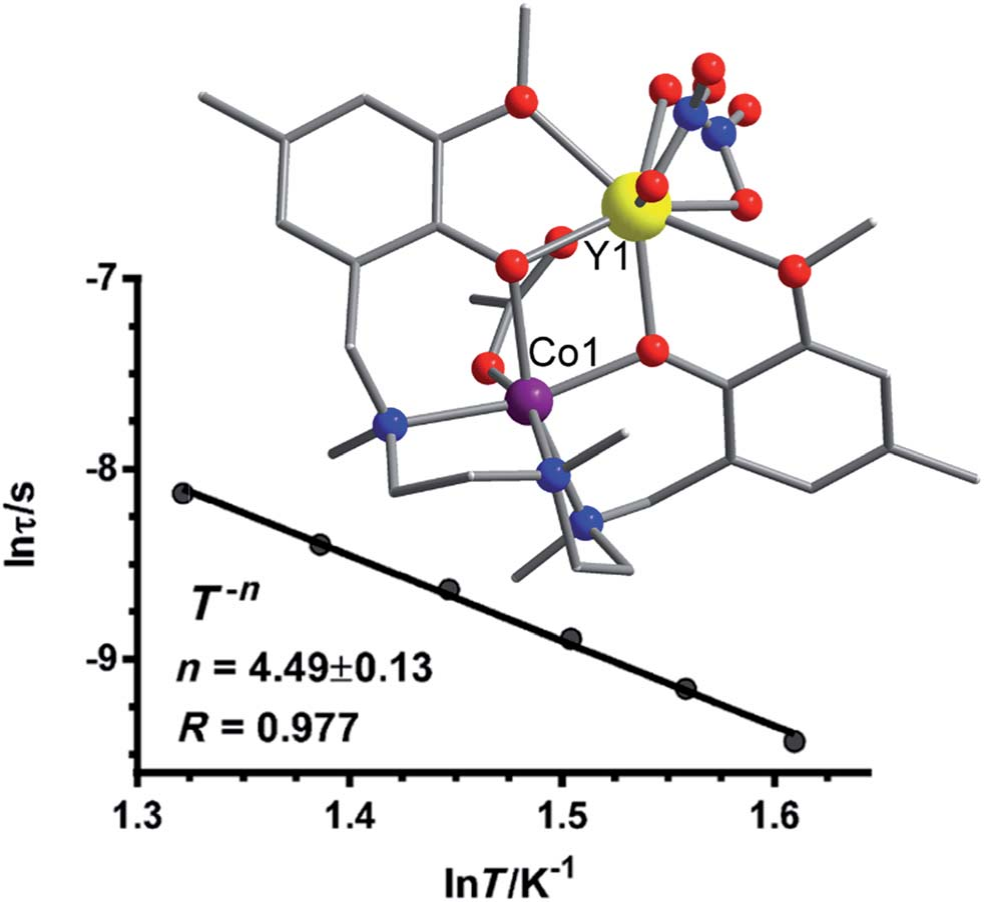
Molecular structure of [Co(L)(OAc)Y(NO_3_)_2_] (**65**), with fitting of the relaxation time to a *T*^–*n*^ power law as described in the text. Colour code: purple (Co), yellow (Y), blue (N), red (O), grey (C). Hydrogen atoms omitted for clarity. Reprinted with permission from ref. 71. Copyright (2013) Wiley-VCH.

Another explanation of slow relaxation and SIM behaviour in a complex exhibiting positive magnetoanisotropy has been given by Colacio and co-workers.^71^ The authors reported a Co(ii)–Y(iii) complex, [Co(L)(OAc)Y(NO_3_)_2_] (**65**) (Fig. 22) where L is the compartmental ligand *N*,*N*′,*N*′′-trimethyl-*N*,*N*′′-bis(2-hydroxy-3-methoxy-5-methylbenzyl)-diethylenetriamine, which forces the Co(ii) ion to adopt trigonally distorted octahedral geometry. Fitting of the ac susceptibility data collected in a 1000 Oe dc field yielded *U*_eff_ = 22.6 K. Dilution studies with a diamagnetic Zn(ii) analogue did not result in an experimentally increased relaxation rate, and the *E* value of the complex was determined to be negligible. As a result the authors were able to rule out phonon bottlenecks and transverse anisotropy respectively, as the causes of the observed slow relaxation. Instead, they suggest that an optical/acoustic Raman process is responsible for the spin relaxation, since the relaxation time (*τ*^–1^) for the complex can be fitted to a *T*^–*n*^ law.^10^ This yields a best-fit *n* = 4.5. The reader should note that in general for Raman relaxation in a Kramers ion, *n* is expected to be 9, however, when both acoustic and optical phonons are considered lower *n* values are routinely obtained. We note that fitting of experimental data to equations of this form is gradually becoming commonplace in the literature, as well as the expanded equation:

4 *τ*^–1^ = *AH*^*m*^*T* + *CT*^*n*^ + *τ*_0_^–1^ exp(–*U*_eff_/*KT*)

Where the parameters *A*, *C* and *τ*_0_^–1^ are constant and the three terms correspond to the direct, Raman and Orbach processes respectively that contribute simultaneously to *τ*^–1^.^10^ This reflects an increasing desire of researchers to rationalise the contributions of different relaxation processes to *U*_eff_.

Luis and co-workers recently focused their attention on the origins of slow magnetic relaxation in [Co^II^(acac)_2_(H_2_O)_2_] (**66**) (acac = acetylacetonate), which is highly anisotropic, *D* = +57 cm^–1^.^72^ This model system was investigated in order to propose an explanation of why SIM behaviour is observed for systems with positive *D* values. They suggest that slow magnetic relaxation, does not necessarily depend on the sign of *D*, but rather occurs naturally as an extension of Kramers theorem, proposing that the magnetic field dependence observed for all complexes with positive *D* is due to strong electro-nuclear spin entanglement. Using the aforementioned Co(ii) complex, they found that the energy barrier obtained experimentally in the presence of an applied field does not correlate well with the calculated first excitation energy. This is rather puzzling of course since QTM should be suppressed under an applied field and hence the barrier for spin reversal should be equivalent, to the gap between the ground and first excited Kramers doublets. It is noteworthy that in order for an Orbach process to be operative it must involve a real transition from one Kramers doublet to another, the gap for which was calculated to be approximately 130 cm^–1^ in this example. At energies between 0 and 130 cm^–1^, no magnetic levels therefore exist, and thus, the obtained barrier cannot be due to an Orbach process. As discussed in Section 1, spin-lattice relaxation can occur *via* three types of process: direct, Orbach and/or Raman pathways. In the studied Co(ii) complex, below 30 K, direct relaxation is prohibited due to the so-called ‘‘Van Vleck Cancellation’’, which implies that a direct transition within a Kramers doublet cannot occur. Additionally, Orbach relaxation is unlikely to occur since the ZFS is greater than *K*_B_*T*, leaving only Raman processes as a possibility for spin-lattice relaxation. Yet, direct processes do occur at low temperatures and, in fact, they dominate below 3 K in this case. This can be explained by hyperfine interactions with the *I* = 7/2 nuclear spin states of Co(ii), generating 16 electro-nuclear spin states, through which spin relaxation may occur. Based on the arguments outlined by the authors explaining the observation of SIM behaviour in Kramers ions with positive anisotropy, we see that another consideration emerges for the design of high-performance SIMs. In addition to half-integer spin and large magneto-anisotropy, the existence of hyperfine interactions must be minimised in order to inhibit direct relaxation processes.

**Fig. 23:**
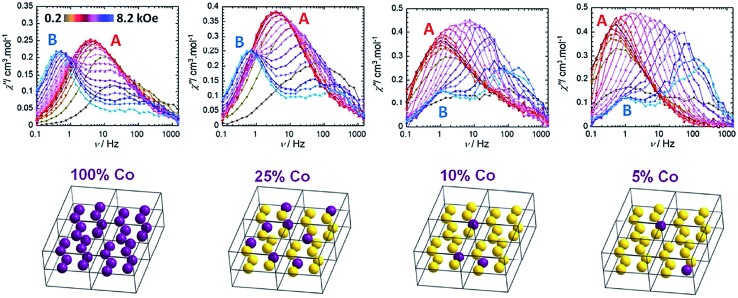
Frequency dependent out-of-phase magnetic susceptibility (*χ*′′) as a function of applied field (200–8200 Oe) at 2 K of [Co(DAPBH)(NO_3_)(H_2_O)] (**67**) magnetically diluted with [Zn(DAPBH)(NO_3_)(H_2_O)]. **A** and **B** represent two relaxation processes. Colour code: purple (Co) and yellow (Zn). Reprinted with permission from ref. 73. Copyright (2015) Royal Society of Chemistry.

We too have recently become interested in the study of easy-plane anisotropy and SIM behaviour in Co(ii) systems.^73^ We turned our attention to the already known 7-coordinate complex, [Co(DAPBH)(NO_3_)(H_2_O)] (**67**) (DAPBH = 2,6-diacetylpyridinebis(2′-pyridylhydrazone)), and systematically doped the sample with varying concentrations of Zn(ii), in order to study the nature of the relaxation processes occurring under an applied dc field (Fig. 23). In particular, our measurements on the pure Co(ii) analogue under a 3.78 Oe oscillating field, in the presence of a static dc field (ranging from 0 to 8200 Oe) at 2 K, revealed the presence of a frequency dependent relaxation process in the out-of phase component of the ac susceptibility (process **A**). As the applied field is increased the observed process shifts to lower frequency reaching its minimum at 1000 Oe, and upon further increasing the applied field, a shoulder peak begins to appear at lower frequency. This starts at 2800 Oe and increases in intensity as the magnitude of the applied field is increased (process **B**). This behaviour, which is clearly indicative of two distinct relaxation processes, has been documented in several other Co(ii) systems, however by doping with Zn(ii) we were able to monitor the ratios of the two processes occurring under different concentrations of Co (100%, 25%, 10% and 5%). In particular, we noticed that process **A** became increasingly dominant at lower Co(ii) concentrations whist process **B** decreases significantly. This data provides clear evidence to support the conclusion that **B** is an intermolecular process rather than an inherent property of the Co(ii) metal centres. This observation has important consequences for researchers studying the dynamic properties of SIM and SMM systems in applied dc fields – one must be acutely aware that magnetic ordering can occur due to intermolecular interactions brought on by large dc fields, yielding ac signals which the uninitiated may incorrectly attribute to a molecular process.

In closing, although not an SIM, we would like to draw attention to a very recent report of a tetrahedral Se-ligated cluster, [Ni{*^i^*Pr_2_P(Se)NP-(Se)*^i^*Pr_2_}_2_] (**68**), studied by Kyritsis, Bogani and Neese, exhibiting easy-plane anisotropy.^74^*D* and *E* values of 45.40 and 1.91 cm^–1^ respectively are reported. What is interesting to us though is that the authors have characterised their system using far-infrared magnetic spectroscopy (FIRMS), a highly useful, but as yet under utilised, technique for directly measuring the ZFS of systems, whose splitting lies outside the frequency range available in commercial HF-EPR instrumentation (∼1 THz or 33 cm^–1^).^75^

## 9. Conclusions and outlook

The field of d-block SIM chemistry is still in its infancy with less than 70 published examples of such systems. Nevertheless, useful conclusions can already be drawn from this body of literature as it stands. In particular, it is striking to note that of the compounds presented in this perspective only nine are SIMs in zero-field. One strategy to rectify this might be to design ligand-field environments, which preserve strict axial symmetry around the chosen metal ion, thus minimising transverse anisotropy and the contribution of QTM to *U*_eff_. The stand-out example of this so far has to be the linear Fe(i) compound (**17**, *U*_eff_ = 325.2 K) of Long and co-workers. Of course, as we have seen, this strategy is by no means guaranteed to work, as relaxation pathways other than QTM are more often than not operative in SIM systems. This brings us conveniently to our next point.

It is clear from surveying the literature that there is currently a strong emphasis within the field being placed on developing strategies to maximise magnetic anisotropy. Whilst this approach of course has merit, emerging literature focussing on high-resolution spectroscopic studies and systems with easy-plane anisotropy suggests that this should not be the sole design criterion when attempting to create high-performance SIMs. We are inclined to agree. In our opinion the exclusive focus on anisotropy is a progress trap, akin to the earlier preoccupation with high spin ground states. If we are truly going to make progress towards the use of SIMs in real applications, then it is important we take a holistic approach that simultaneously considers not only spin and anisotropy (linked to the ligand field), but also how the magnetic moments of individual ions interact with their environment (the crystal lattice). In other words, we need to gain insight into how we can control the contributions of Orbach, Raman and direct relaxation processes to *U*_eff_. In this respect, the inter-disciplinary nature of molecular magnetism is now more important than ever, as dealing with these challenges will require the concerted effort of synthetic chemists, experimental spectroscopists and theoreticians.

From a synthetic chemistry point of view, one obvious area of future exploration is the synthesis of 4d and 5d SIM systems. The spin–orbit coupling constants of second and third row transition metal ions are inherently larger than their first row counterparts, potentially leading to improved SIM properties. Indeed, there is already a small body of literature concerning the synthesis of Re(iv) based compounds.^76^ In addition, the increased radial extension of the 4d/5d orbitals allows for stronger exchange interactions, an important consideration in the design of single-chain magnets. Other as yet unexploited candidates for 4d/5d SIM systems include Nb(iii) and Ru(iii).

Finally, if the SIM (and by extension SMM) field is to move forward constructively, then it is important that researchers remain cautious in their interpretation of results and rigorous in their magnetic characterisation. As we (and others) have demonstrated, the application of large dc fields to supress QTM in ac susceptibility studies can lead to the appearance of frequency dependent peaks, which are not the result of molecular processes but rather a result of field-induced intermolecular interactions.

Regardless of how the field develops over the next few years however, one thing is certain – after several years spent in the shade at the behest of their lanthanide cousins, 3d metal ions of the first-row have well and truly come back to the fore.
